# Neurobiological research on *N,N*-dimethyltryptamine (DMT) and its potentiation by monoamine oxidase (MAO) inhibition: from ayahuasca to synthetic combinations of DMT and MAO inhibitors

**DOI:** 10.1007/s00018-024-05353-6

**Published:** 2024-09-10

**Authors:** Klemens Egger, Helena D. Aicher, Paul Cumming, Milan Scheidegger

**Affiliations:** 1 Department of Adult Psychiatry and Psychotherapy, Psychiatric University Clinic Zurich and University of Zurich, Zurich, Switzerland; 2 Neuroscience Center Zurich, University of Zurich and Swiss Federal Institute of Technology Zurich, Zurich, Switzerland; 3 Department of Nuclear Medicine, Bern University Hospital, Bern, Switzerland; 4 Department of Psychology, University of Zurich, Zurich, Switzerland; 5 School of Psychology and Counselling, Queensland University of Technology, Brisbane, Australia

**Keywords:** Psychedelics, Ayahuasca, DMT, Harmine, β-carbolines, MAO inhibition

## Abstract

The potent hallucinogen *N,N-*dimethyltryptamine (DMT) has garnered significant interest in recent years due to its profound effects on consciousness and its therapeutic psychopotential. DMT is an integral (but not exclusive) psychoactive alkaloid in the Amazonian plant-based brew ayahuasca, in which admixture of several *β*-carboline monoamine oxidase A (MAO-A) inhibitors potentiate the activity of oral DMT, while possibly contributing in other respects to the complex psychopharmacology of ayahuasca. Irrespective of the route of administration, DMT alters perception, mood, and cognition, presumably through agonism at serotonin (5-HT) 1A/2A/2C receptors in brain, with additional actions at other receptor types possibly contributing to its overall psychoactive effects. Due to rapid first pass metabolism, DMT is nearly inactive orally, but co-administration with *β*-carbolines or synthetic MAO-A inhibitors (MAOIs) greatly increase its bioavailability and duration of action. The synergistic effects of DMT and MAOIs in ayahuasca or synthetic formulations may promote neuroplasticity, which presumably underlies their promising therapeutic efficacy in clinical trials for neuropsychiatric disorders, including depression, addiction, and post-traumatic stress disorder. Advances in neuroimaging techniques are elucidating the neural correlates of DMT-induced altered states of consciousness, revealing alterations in brain activity, functional connectivity, and network dynamics. In this comprehensive narrative review, we present a synthesis of current knowledge on the pharmacology and neuroscience of DMT, *β*-carbolines, and ayahuasca, which should inform future research aiming to harness their full therapeutic potential.

**Supplementary Information:**

The online version contains supplementary material available at 10.1007/s00018-024-05353-6.

## Introduction

Because of their profound effects on the human mind, psychedelic substances have been the object of fascination in the Western world since the 1950s [1], when Humphrey Osmond coined the term psychedelic. Despite pioneering work by Alexander and Ann Shulgin on the synthesis and subjective effects of phenylethylamines and tryptamines [2, 3], a long-standing moratorium on funding of psychedelics research impeded progress in understanding basic aspects of the physiology and phenomenology of psychedelic substances. In this narrative review, we summarize the state of knowledge of *N,N*-dimethyltryptamine (DMT), which has been used for millennia by indigenous peoples of South and Mesoamerica for healing and spiritual purposes in the form of the herbal brew variously known as yajé or ayahuasca [4, 5].^1^ Uniquely, ayahuasca brew often contains a mixture of DMT along with several *β*-carboline alkaloids, which together enhance the bioavailability of orally administered DMT by blocking its first pass metabolism by monoamine oxidase A (MAO-A) in the gut and other organs. Whereas oral DMT alone is nearly inactive, DMT is potently psychoactive when inhaled as vapor [6], and when taken via intravenous administration [7, 8], i.e., routes that circumvent the first-pass metabolism.

The classical psychedelics lysergic acid-*N,N*-diethylamide (LSD) [9] and psilocybin (prodrug of the psychoactive substance psilocin) are agonists or partial agonists at 5-hydroxytryptamine (serotonin) 2A (5-HT_2A_) receptors in brain [1], which are the key mediators of their psychedelic effects. While DMT is generally included among the classical psychedelics, as shall emerge below, it is not yet certain that 5-HT_2A_ receptor agonism exclusively mediates DMT/ayahuasca effects. Investigations of ayahuasca’s pharmacological effects and therapeutic potential are at a relatively nascent stage, mainly confined to its use in naturalistic and traditional settings.

Like classical psychedelics, consumption of ayahuasca leads to profound alterations in consciousness, characterized by changes in perception and the “inner (cognitive and emotional) experiences” [5, 10, 11], with “visuals, kaleidoscopic lights, geometrical forms, tunnels, animals, humans and supernatural beings coinciding with sensations of peace, harmony and inner calm” [12]. Other commonly experienced phenomena include synesthesia [13], decentered introspective states [14], emotional release [15], attribution of meaning [14, 16], alterations in meaningful, guiding values in life [14, 17], ego dissolution, better understanding of oneself and others, acceptance of oneself and past life events [14, 18], and expansive states with transpersonal experiences [19]. Unlike other psychedelics, ayahuasca effects also include notable physical sensations like nausea and vomiting, which may be integral to its traditional use in healing and spiritual rituals [20]. Indigenous and neo-shamanic groups attribute transformative healing properties to the spirit of ayahuasca, often experienced through vivid encounters with plant spirits in a culturally rich ritual setting [21].

Recent pre-clinical and observational studies have shown encouraging results with ayahuasca in treating a variety of conditions and their animal models, including depression, anxiety, PTSD [22–33], substance use disorders [34–38], eating disorders [39, 40], and grief [41, 42]. In an initial clinical trial, ayahuasca has shown efficacy against depression and anxiety symptoms [28] and in altering brain network dynamics linked to depression pathophysiology [43]. In a randomized placebo-controlled trial (RCT) conducted in Brazil, a single ayahuasca dose produced rapid antidepressant effects persisting for weeks in (n = 29) patients with treatment-resistant depression [30]. In a recent observational study, the majority of (n = 20) individuals with initial diagnosis of major depression disorder (MDD) enjoyed remission lasting a year after their participation in a ritual with administration of botanical ayahuasca analogues (i.e., various plant sources of DMT and *β*-carbolines) in the context of an ayahuasca ritual [32]. Preclinical and in vitro investigations suggest that ayahuasca chemical constituents may also possess neuroprotective properties in neurodegenerative disease models [44–46]. Thus, a comprehensive review of 21 clinical and preclinical studies with chemical constituents of ayahuasca revealed consistent findings of anxiolytic, antidepressant, anti-addictive, and neuroprotective properties [47].

Psychological support is critically important during a therapeutic ayahuasca experience, given the influence of contextual factors on mental health outcomes [48]. The burgeoning interest in ayahuasca's therapeutic benefits marks a pivotal shift from traditional to clinical contexts, opening new avenues for research and application in Western medicine. Its uniquely complex blend of pharmacological, psychological, and cultural elements makes ayahuasca an intriguing research area for scientists from various disciplines. Our objective in this narrative review is to bridge the gap between the phenomenology of the ayahuasca experience and western models of neuropharmacology and brain function. Therefore, we have compiled the current state of knowledge of the pharmacology, biochemistry, and neuroscience of DMT, emphasizing its synergism with MAOIs in the contexts of ayahuasca and its botanical and synthetic analogs. We first summarize the historical and cultural background of ayahuasca, and then elaborate upon the known pharmacological, molecular, cellular, and functional mechanisms of action of the DMT/MAOI combination from studies in vitro and imaging studies in vivo.

## Ayahuasca: traditional botanical forms

Ayahuasca (also known as yajé, hoasca, etc.) is a Hispanicized term borrowed from Quechuan dialects of the Amazon basin, which refers to the woody vine (liana) *Banisteriopsis caapi* and its decoctions, as used for ritual and healing purposes [5]. The psychoactive beverage is prepared by extensive boiling of the *B. caapi* bark, resulting in a thick, brown, and oily liquid [49]. The prolonged boiling process is necessary to extract the plants’ alkaloids, which have low solubility in water. Indeed, the *β*-carboline harmine mainly resides in the solid phase of the ayahuasca brew [50]. Recipes for traditional ayahuasca differ between indigenous peoples and geographic regions [51]. Some traditional shamanic rituals using ayahuasca as a sacred medicine employ decoctions mainly from *B. caapi*, which contains *β*-carboline MAOIs, but little or no DMT. Traditional ayahuasca decoctions often contain DMT derived from the leaves of plants such as *Psychotria viridis, P. carthagenensis,* or the amazonian shrub *Diplopterys cabrerana* [52, 53]*.* In popular conception, the *B. caapi* MAOIs serve only to enhance the bioavailability of DMT derived from other ayahuasca components. However, DMT-containing plants are not always included in ayahuasca brews; some indigenous groups in the Amazon basin use *B. caapi* alone for initiation or healing practices*,* without admixture of any other plant material [54, 55]. Furthermore, some ayahuasca decoctions contain tobacco or other psychoactive plants [56]. Nonetheless, we suppose that a binary DMT/MAOI model may best capture the complex ayahuasca experience that derives from ancient traditional knowledge of indigenous people who have used these brews in one form or another since millennia [57].

The essential ayahuasca component *B. caapi* contains several *β*-carbolines from the harmala alkaloid family of tryptophan metabolites [58], which may be psychoactive in their own right [59], in addition to their inhibition of DMT metabolism by MAO-A [60]. The various *β*-carbolines in *B. caapi*, especially harmine and harmaline, enable the attainment of sufficient plasma DMT concentrations to evoke psychedelic effects lasting 4–6 h [5, 61]. Re-dosing four hours after the first ayahuasca administration prolongs the subjective effects, likely due to accumulation of alkaloid concentrations in the body [62] (repeated dosing is typical of traditional ayahuasca rituals). Tetrahydroharmine (THH), the second-most abundant *B. caapi β*-carboline, is also a weak inhibitor of plasma membrane serotonin transporters (SERT) [63], i.e., the site of action of selective serotonin reuptake inhibitor (SSRI) antidepressants. THH may also contribute to net MAO inhibition despite its weaker affinity as compared to harmine and harmaline [10, 64]. The *B. caapi β*-carbolines are almost exclusively MAO-A inhibitors, with 100-fold lower affinity for MAO-B [65, 66]. However, it is by no means certain that DMT and *β*-carbolines are the only pharmacologically relevant compounds in ayahuasca; the chemical diversity in the plant matrix predicts an “entourage effect” [67] that remains uninvestigated. For the present, we focus on the most abundant ayahuasca *β-*carbolines (harmine, THH and harmaline) and their interactions with DMT [68].

### *β*-carbolines and DMT concentrations in ayahuasca samples

We summarize in Table 1 findings of studies reporting concentrations of harmine, harmaline, THH, and DMT in ayahuasca samples from different geographical and indigenous origins. In considering the results of these field sample studies, there is clearly no standard alkaloid composition or standard dose, and that factors such as quantity and quality of used plants, the geographic region, and likewise the cultural affiliations of the people producing ayahuasca all contribute to its varying composition [69]*.* The rank order of *β*-carboline concentrations is generally harmine ≥ THH > harmaline, where harmine concentrations tended to only slightly exceed the THH concentrations, and harmaline was overall the least-abundant *β*-carboline alkaloid. Indeed, the reported concentrations range from 0.06 to 22.9 mg/mL harmine, 0–1.72 mg/mL harmaline, 0.02–23.8 mg/mL THH and 0.05–14.2 mg/mL DMT (Table 1). Despite considerable variability, the analytical findings generally predict that one cup (200 mL) of typical ayahuasca brew would contain alkaloid doses up to a few hundred mg. During the extended boiling process of ayahuasca preparation, harmine converts via consecutive reduction reactions to harmaline and then to THH, thus shifting the *β*-carboline ratios as compared to the untreated *B. caapi* [70]. Furthermore, THH is more chemically stable than harmine/harmaline, surviving in ayahuasca stored for nine days at 37 °C [71]. Variable DMT concentrations likely reflect the proportion of *P. viridis* to the total plant material, which ranged from 7 to 20%, depending on the preparation recipe [70]. It remains unknown if alkaloid concentrations in *B. caapi* differ across geographic regions or depending on season of harvest.

**Table 1:** Concentrations of the main ayahuasca alkaloids harmine, harmaline, tetrahydroharmine (THH) and *N,N-*dimethyltryptamine (DMT) in different ayahuasca samples

	Harmine		Harmaline		THH		DMT		Sample size	Analytical method	Origin
	Concentration mg/mL	% of total alkaloid content	Concentration mg/mL (or mg/g)	% of total alkaloid content	Concentration mg/mL (or mg/g)	% of total alkaloid content	Concentration mg/mL (or mg/g)	% of total alkaloid content			
Rivier and Lindgren [72]	0.06–0.19	21.0–62.0	0–1.6 × 10^–2^	0.0–4.0	1.5 × 10^–2^–9.8 × 10^–2^	6.0–40.0	5.4 × 10^–2^–1.6 × 10^–1^	0.0–41.0	9	GC–MS	Various Peruvian ayahuasca tribes
McKenna et al. [52]	3.40–5.51	53.0–67.0	0.30–0.51	5.0–6.0	1.06–1.94	20.0–30.0	0.51–0.70	6.0–11–0	5	HPLC	Peruvian shamans, undiluted samples
	8.60–57.6 (mg/g)	27.0–76.0	4.20–6.30 (mg/g)	4.2–6.3	8.00–25.5 (mg/g)	10.5–38.0	0–7.2 (mg/g)	0.0–7.2	5	HPLC	Peruvian shamans, lyophilized samples
Callaway et al. [73]	0.45–22.9		0–0.90		0.45–23.8		0–14.2		29	HPLC-FD	Barquinha, Santo Daime, UDV from Brazil; Shuar from Ecuador
Pires et al. [74]	0.37–0.83		0.64–1.72		0.21–0.67		0.31–0.73		8	GC-NPD	religious group settled in Araçoiaba da Serra, Brazil
Souza et al. [75]	0.41–1.82		0.04–0.42		0.04–3.31		0.06–0.34		38	LC–MS/MS	UDV centers, Brazil
Lanaro et al. [76]	0.30–2.89		0.03–0.18		0.85–2.05		0.40–2.07		9	HPLC–DAD	Center of Integral Development Luz do Vegetal, Brazil
Santos et al. [70]	0.11–7.11		0.01–0.95		0.09–3.05		0.10–3.12		33	LC–MS/MS	Santo Daime or UDV in Brazil
Kaasik et al. [53]	0.14–4.44	15.0–75.0	4 × 10^–3^–0.39	0.3–16.8	0.03–3.88	1.4–55.4	0.09–2.69	9.2–63.7	99	UHPLC-MS/MS	worldwide; Shamanic & neoshamanic, as well as ayahuasca churches
Rodríguez et al. [50]	1.34–5.61 (mg/g)		0.16–0.45 (mg/g)		1.03–2.33 (mg/g)		0.53–1.86 (mg/g)		6	qNMR	Uruguay; religious Brazilian churches, neoshamanic practitioners or holistic centers

Note that percentages must not necessarily come from the same min/max sample as the concentration values

Abbreviations: *GC–MS* gas chromatography–mass spectrometry, *GC-NPD* gas chromatography–nitrogen–phosphorous detector, *LC–MS/MS* liquid chromatography–tandem mass spectrometry, *(U)HPLC–MS/MS* (ultra) high performance liquid chromatography–tandem mass spectrometry, *HPLC–DAD* high performance liquid chromatography–diode-array detector, *HPLC-FD* high performance liquid chromatography–fluorescence detection, *qNMR* quantitative nuclear magnetic resonance

## Ayahuasca analogues and pharmahuasca

The eponymic harmala *β*-carboline alkaloids in *B. caapi* also occur in plants such as *Peganum harmala* (Syrian rue), which is native to Eurasia and northern Africa, or the flowers of the mainly American *Passiflora incarnata* (passionflower). DMT in ayahuasca often derives from plants of genera *Psychotria*, the Brazilian/Mesoamerican *Mimosa hostilis* (jurema), or *Anadenanthera and Diplopterys* [53, 77]. The ubiquity of these alkaloids likely reflects their derivation from the amino acid tryptophan, but there is evidence that tryptamine alkaloids confer increased resistance against herbivores or other predators [78, 79]. Brews comprising plant sources other than *B. caapi* and *Psychotria* are ayahuasca analogues, whereas synthetic formulations are commonly known as “pharmahuasca” [53, 56]. Ayahuasca analogue formulations commonly include *P. harmala* as a *β*-carboline source and *M. hostilis* or *A. confusa* as a DMT source [53, 56, 80]. *P. harmala* (mainly its seeds) has traditional medicinal uses in Iran [81] for its supposed cardiovascular, neurologic, antimicrobial, gastrointestinal (GI), and antidiabetic effects [82], and *M. hostilis* finds use in South and meso-American spiritual and shamanic rituals [83, 84].

In the 1960s, Claudio Naranjo reported on the use of harmaline for Western psychotherapy [85], highlighting its potential therapeutic benefits for facilitating introspection, emotional release, self-awareness, and personality integration. It remains uncertain if such effects derive from MAOI or other pharmacological properties of harmaline. Advancements in DMT synthesis and the broader availability of pharmaceutical MAOIs were drivers for the increasing popularity of pharmahuasca. Particularly in Europe, ayahuasca analogues and pharmahuasca are often less costly and more accessible than authentic ayahuasca [53, 86, 87]. Furthermore, uncontrolled harvesting of *B. caapi* is a recognized threat to its viability in the wild [88]. While ayahuasca analogues and pharmahuasca can produce experiences akin to traditional ayahuasca, their specific effects differ according to the alkaloid composition [86, 87]. Synthetic formulations potentially offer more standard alkaloid composition and a better safety profile, notably with respect to the occurrence of emesis (a”purge” is considered an essential and therapeutic aspect of the ayahuasca ritual) [89]. Indeed, having a standard composition remains a key requirement for inclusion of medicine in an approved Western pharmacopeia, although there is not yet a consensus on the optimal composition of ayahuasca alkaloids.

## *N,N-*dimethyltryptamine (DMT)

**Fig. 1:**
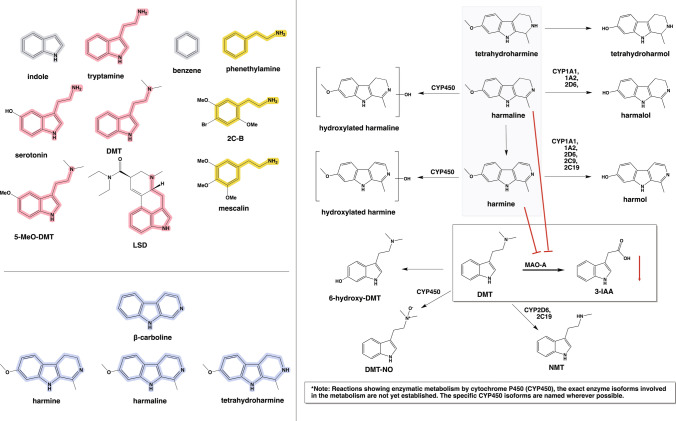
Molecular structures of *N,N-*dimethlytryptamine (DMT) and other psychedelics, the main ayahuasca *β*-carbolines, and key metabolic pathways. **A** Indole and benzene rings (gray) are the chemical scaffolds of the two main categories of psychedelics, i.e. tryptamines (red) and phenethylamines (yellow). Serotonin, DMT and 5-MeO-DMT are structurally similar; LSD, while also containing the tryptamine (and phenethylamine) motif, is an ergoline derivative. Among the phenethylamine psychedelics, we present the structures of 4-bromo-2,5-dimethoxyphenethylamine (2C-B) and mescaline. **B** The *β*-carboline scaffold of harmine, harmaline, and tetrahydroharmine (THH) are shown in blue. **C)** These main *β*-carbolines in ayahuasca undergo demethylation to harmol, tetrahydroharmalol, and harmalol, respectively. Several cytochrome (CYP) enzymes are implicated in the demethylation of harmine and harmaline, but details are lacking for THH. Harmine and harmaline can also undergo ring-hydroxylation catalyzed by CYP450 [107, 108]. An additional metabolic route of harmaline is its oxidation to harmine. DMT is predominantly metabolized by oxidative deamination via monoamine oxidase type A (MAO-A), followed by formation of indole-3-acetic acid (3-IAA) by non-specific aldehyde dehydrogenases. Alternately, DMT is oxidised to DMT-*N*-oxide (DMT-NO) by CYP450 or demethylated by CYP2D6 and CYP2C19 to *N-*methyltryptamine (NMT), or hydroxylated to 6-hydroxy-DMT by yet unknown enzymes [60, 107, 109, 110]. Red arrows indicate inhibition of DMT metabolism by the *β*-carboline MAO-A inhibitors, resulting in lesser formation of 3-IAA

DMT derives from tryptamine, which forms by decarboxylation of *L*-tryptophan catalyzed by the enzyme aromatic amino acid decarboxylase (AAADC; commonly known as DOPA decarboxylase) (Fig. 1). As first described by Axelrod [90], DMT biosynthesis proceeds by a two-step process from tryptamine via the enzyme indolethylamine *N*-methyltransferase (INMT), a transmethylation enzyme using *S*-adenosyl-*L*-methionine (SAM) as methyl donor. The product *N*-methyltryptamine (NMT) undergoes further methylation by the same enzyme to give DMT. In situ hybridization studies revealed expression of INMT in neurons, co-localizing with DOPA decarboxylase in presumably DMT-synthesizing neurons in cerebral cortex, and in choroid plexus, but with highest concentration in lung tissue [91, 92]. However, INMT knockout in a rodent model, failed to ablate tryptamine methylation in brain and lung tissue, suggesting the presence of alternate enzymatic pathways [93]. DMT is present in many mammalian tissues. Indeed, the interstitial DMT concentration in rodent brain was approximately 1 nM to cerebral microdialysis coupled with HPLC [92]. Cerebral microdialysis analysis of canonical biogenic monoamine neurotransmitter concentrations (e.g., serotonin, dopamine, norepinephrine) showed similar concentrations in the range of ~ 1–4 nM [94]. UHPLC-MS analysis of brain tissue extracts indicated DMT concentrations ranging from zero to 30–60 nM [95, 96]. The detection of DMT in the pineal gland [97] inspired the concept that pineal DMT release might induce vivid dreams, or near-death and other mystical-type experiences [98], but the total quantity of pineal DMT seems insufficient to evoke such effects. Studies of endogenous DMT concentrations in body fluids (mainly blood and urine) are generally uninformative about the cellular sites of DMT production in biologically significant amounts [98].

Exogenous DMT rapidly accumulates in the rat brain after i.p. or i.v. administration, transiently attaining a brain:blood partition ratio of approx. 5–6:1, followed by rapid clearance from the brain and circulation [96, 99–101]. Nonetheless, DMT remained detectable in the rabbit CNS up to seven days after peripheral administration, while urinary excretion was not detectable after 24 h [102], which could be consistent with storage in a very stable vesicular pool. After i.p. administration, there was DMT accumulation in the cerebral cortex, amygdala, and caudate-putamen, while medulla oblongata and cerebellum only showed low uptake [101], suggesting compartmentation within specific neuronal populations. We have reported spatially heterogeneous DMT accumulation in rat brain after i.p. administration, with 50% higher concentrations in the frontal cortex than in the cerebellum [103], again suggesting some mechanism for its retention in brain tissue. Indeed, DMT can enter serotonin neurons via SERT, and then accumulate in synaptic vesicles as a substrate for the vesicular monoamine transporter 2 (VMAT2) [104]. Storage in a vesicular compartment would protect DMT from MAO degradation, and might support its release from serotonin fibers as a “false neurotransmitter” [101]. To qualify as a classical neurotransmitter, an endogenous substance must be present in physiologically significant amounts, with release in a calcium-dependent manner after presynaptic depolarization, and then evoking responses at specific post-synaptic sites [105]. Given the current evidence, endogenous DMT may meet these criteria [106], despite its low affinity at 5-HT_2A_ receptors. For an extensive discussion of DMT as a candidate neurotransmitter, see [106].

## MAO inhibitors

MAO enzymes (enzyme commission number EC 1.4.3.4) are amine oxidoreductases, with main expression in the outer mitochondrial membrane of mammalian cells. MAO substrates include the biogenic monoamine neurotransmitters dopamine, epinephrine, norepinephrine, and serotonin, and the exogenous psychedelics DMT, psilocin and mescaline. The MAO-reaction consumes molecular oxygen in the restoration of the reduced FADH_2_ cofactor to its active FAD form; the imine intermediate spontaneously eliminates ammonia, and the resultant aldehyde is oxidised to the carboxylic acid by non-specific NAD + -dependent dehydrogenase enzymes [111]. The two isoforms of MAO, which arose from a gene duplication event, have very similar amino acid sequences [112], but somewhat distinct primary substrates. Whereas serotonin and DMT are preferred substrates for MAO-A, phenylethylamine is a MAO-B substrate; both isozymes metabolize dopamine and tyramine with little selectivity [113, 114]. MAO-A occurs in the brain, GI tract, liver, the vasculature of the lungs, as well as in the placenta, while MAO-B mainly occurs in blood platelets [111], astrocytes [115], and certain specific populations of neurons [116]. With respect to ayahuasca, MAO-A in the GI tract is the principal determinant of DMT absorption.

Whereas harmine and moclobemide are reversible MAO-A inhibitors, certain propargyl compounds form a covalent bond with the enzyme, rendering it permanently inactive. The non-selective irreversible MAOIs phenelzine, isocarbaxazid, and tranylcypromine emerged in the mid-twentieth century as the first effective pharmacotherapeutic agents for depression [117]. These medications have since largely fallen out of favor due to the perceived risk of interactions with dietary vasoactive amines (the”cheese effect”) or the serotonin syndrome, a potentially fatal crisis of hypertension, fever, delirium, and rhabdomyolysis that can occur upon co-administration of direct or indirect serotonin agonists. As such, irreversible MAOIs now seldom serve as first or second line antidepressants, but remain in use in certain severe and treatment-resistant cases, which calls for strict observation of dietary restrictions [118]. However, serotonin syndrome and hypertensive crisis are exceedingly rare events in patients treated with irreversible MAO blockers [118].

The reversible MAO-A inhibitor moclobemide is an antidepressant with some efficacy in treating social anxiety, being notable for its favorable side-effect profile and relatively brief plasma half-life. Moclobemide has occasionally been detected in neo-shamanic recipes in Europe [53]. In general, pretreatment with any inhibitor of MAO-A, reversible or irreversible, would likely serve for potentiation of DMT bioavailability after oral administration, we are not aware of MAOIs other than harmine and moclobemide finding use in pharmahuasca.

## Safety and risks associated with ayahuasca or DMT use

Despite the theoretical risk of serotonin syndrome, there are preclinical reports showing potentiation of DMT effects by co-administration of irreversible MAO inhibitors iproniazid or pargyline treatment [119, 120]. In an ayahuasca neurotoxicity study, some rats showed behavioral signs of serotonin syndrome and eventually died after receiving doses some 30- and 50-fold the typical human doses [121]. However, only at such extreme doses can the reversible MAOIs in ayahuasca (or generally also pharmahuasca) evoke the nearly complete inhibition that may be a precondition for the serotonin syndrome. Observational studies have not raised major safety concerns for ayahuasca practitioners taking SSRIs [122].

Neither short-term nor long-term ayahuasca use led to dependency, and its use in controlled settings such as ceremonial contexts suggests an acceptable safety profile [49, 76, 123]. Acute treatment-emergent adverse events (TEAEs), mainly nausea and vomiting (69.9%), typically resolved without an intervention, with few (2.3%) such participants needing medical attention [124, 125]. The American National Poison Data System (NPDS) registered 538 adverse events for ayahuasca between 2005 and 2015, with 28 cases requiring intubation, four cases of cardiac arrest, 12 seizures, and three fatalities [126]. When considering the global prevalence of ayahuasca use, estimated to be over 4 million annually, the number of deaths (n = 58) reported in association with its use is low. Notably, those fatalities have not been linked to traditional ayahuasca ingredients but may involve toxic plant admixtures, drug interactions, or pre-existing conditions [127].

On the other hand, challenging psychedelic experiences are common (55.9%), with adverse psychological reactions typically subsiding within a few days; however, 12% of such individuals sought additional professional support [124, 125]. Severe psychological distress, including severe depression and psychotic episodes, can occur with ayahuasca use [128, 129]. Contemporary neo-shamanic and tourist-oriented settings therefore adopted a broad spectrum of general safety and good practice guidelines. However, some participants in contemporary ayahuasca rituals may lack adequate cultural support and guidance [129, 131]. Traditional indigenous settings usually provide structure and safety within ancestral medicinal practices (e.g. plant dietas) contemporary touristic settings. While certain structured approaches like specific dietary protocols, careful attendance, and setting might mitigate risks and enhance the experience [130], the Western concept of psychological support may not neatly align with such Indigenous methods. Traditional indigenous settings often lack formal health screenings and discussions on medication interactions, challenging the assumption that they are inherently safer for tourists.

Importantly, there is need to integrate safety measures for interactions between ayahuasca with prescription medications (i.e., SSRIs or dopaminergic stimulants), other drugs of abuse, or specific foods rich in tyramines such as overripe fruits, fermented food, tofu, or nuts, which might conceivably increase the risk of serotonin syndrome [11, 128]. The use of ayahuasca is not recommended for individuals with uncontrolled hypertension, cardiovascular or cerebrovascular diseases, epilepsy, glaucoma, and liver or gastrointestinal diseases (e.g. ulcers or gastritis), and during pregnancy [131]. Furthermore, ayahuasca may be risky for individuals with severe psychiatric conditions, including bipolar or psychotic disorders [131].

## Mechanisms of action: ayahuasca and DMT alone

### Pharmacological mechanisms

#### Human pharmacokinetics and pharmacodynamics of DMT and ayahuasca

In the 1950s, the Hungarian chemist and psychiatrist Stephen Szára undertook the first investigations of psychological and hallucinogenic effects of DMT, which he self-administered intramuscularly (i.m.) as an extract from *M. hostilis* [132]. In the 1970s, Dittrich, Bickel, and colleagues presented the first systematic psychological investigations of i.m. DMT administration [133, 134]. Rick Strassmann reported that intravenous (i.v.) DMT at doses ranging from 0.03 to 0.25 mg/kg DMT freebase (as fumarate) induced peak psychedelic effects at five minutes for the 0.25 mg/kg dose, with plasma DMT concentrations peaking at 16 ng/mL (85 nM) [135]. Subjective effects returned to baseline by 30 min. Recent studies tested i.v. DMT with different administration regimens. Such protocols entailed 0–19.2 mg bolus 0.5–0.8 mg/min constant infusion of DMT freebase (as hemifumarate) for up to 90 min (Basel) [7], 11.2 mg bolus 1.2 mg/min infusion of DMT freebase (as fumarate) for up to 30 min (London) [8], and constant infusion totaling 13.4 mg DMT freebase (as fumarate) over 10 min (London) [110]). The Basel study showed dose-dependent increases in heart rate up to 119 BPM and blood pressure up to 159/98 mmHg [7], peaking shortly after the bolus administration and stabilizing within 10–15 min. This aligns with findings from the London study [8], suggesting a good physiological safety margin in individuals without cardiovascular disease or hypertension. In the first randomized controlled trial of a standardized ayahuasca-analogue formulation containing DMT/harmine, oral doses included up to 38.4 mg DMT freebase (as hemifumarate) and 250 mg  harmine, or up to 69.1 mg intranasal DMT freebase (as hemifumarate) [89]. DMT was given in 7.7 mg portions at 15 min intervals intranasally, in combination with buccal harmine (up to 200 mg). Autonomic parameters increased transiently after DMT administration and returned to baseline within 120–180 min, with fewer side-effects (e.g. nausea, headache) compared with botanical ayahuasca. In recent intravenous DMT studies, peak plasma concentrations (C_max_) were 61 ng/mL at T_max_ (2.9 min) [7], 32 ng/mL after 11.2 mg DMT bolus followed by 1.2 mg/min [8], and 63 ng/mL after constant infusion of 1.34 mg/min DMT (freebase weight) [110]. These C_max_ values correspond to DMT concentration range of 170–335 nM, with apparent plasma half-life (t_1/2_) of 5–12 min [8, 109]. In comparison, C_max_ for intranasal DMT (combined with buccal harmine) was 33 ng/mL at 130–140 min after first administration of the highest dose combination [89]. Surprisingly, the intravenous DMT studies revealed large inter-individual variability in plasma concentrations [7, 110]. This is likely due to individual differences in whole body MAO activity, suggesting a need for personalized dosing. The intranasal/buccal routes of administration considerably improved upon the PK variability of combined oral DMT/harmine [89]. However, determining the appropriate extent of MAO inhibition to optimize DMT bioavailabilty, remains challenging due to inter-individual differences in harmine metabolism (i.e. rapid vs. slow metabolizers [136]). Overall, i.v. DMT and parenteral DMT/harmine administration routes can evoke subjective states of controlled intensity and duration, but further refinement of dosing protocols is needed.

The pharmacokinetics of ayahuasca decoctions, which contain a mixture of β-carboline alkaloids, are more complex than for pharmaceutical combinations of DMT and harmine. The presence of THH and harmaline also influence the pharmacodynamics of DMT, while possibly having psychoactive effects unrelated to MAO inhibition. Administration of natural ayahuasca at doses corresponding to 1.4 mg/kg DMT, 4.6 mg/kg harmine, 0.75 mg/kg harmaline and 5.4 mg/kg THH evoked C_max_ values of 25 ng/mL DMT and 110 ng/mL harmine [137], which are comparable with C_max_ values from the highest dose in the DMT/harmine PK study [89]. We suppose that THH, given its C_max_ of 329 ng/mL (1.5 µM) from natural ayahuasca, could well contribute to ayahuasca psychopharmacology. An earlier study with administration of lyophilized ayahuasca capsules reported significant plasma concentrations of DMT and THH, but no detectable harmine and harmaline, despite their presence in the capsules [61]. Indeed, the concentrations of DMT and THH were lower than expected by the authors, based on the ayahuasca PK study conducted earlier by Callaway and colleagues [10]. The authors interpreted the disparate plasma results as reflecting differing bioavailability of alkaloids in the lyophilized capsules as compared to the botanical ayahuasca brew [61]. In another study involving administration of two successive ayahuasca doses at four hours apart, there was substantial potentiation of DMT plasma concentrations (approximately 25% higher C_max_ after the second dose) and subjective effects after the second dose [62]. These results suggest a lack of acute tolerance to subjective effects, and furthermore indicate that carryover of alkaloids from the first dose augments the MAO inhibition from the second dose, which is consistent with the 3–5 h plasma half-lives of harmine and harmaline (40 mg/kg, i.p.) seen in rats [138]. Indeed, repeated dosing schemes are very common in the ayahuasca ritual, with (anecdotally) little or no development of tolerance on a time scale of days. Such a lacking rapid tolerance development contrasts with LSD or psilocybin, which show significantly declining subjective effects when taken on consecutive days, in association with cross-tolerance [139, 140]. On the other hand, the continuous i.v. DMT administration studies reported the strongest subjective effects directly after onset, which subsequently declined despite increasing blood plasma DMT levels over time [7, 8]. Such results imply the occurrence of partial acute short-term tolerance to DMT alone, even though there is a general correspondence between pharmacodynamic subjective effects induced by DMT and ayahuasca with the plasma concentrations of the relevant alkaloids. This holds especially well for plasma DMT curves, which are in good accord with the T_max_ for overall intensity, visual effects, side effects, and other subjective acute effects [8, 10, 61, 89, 110, 137, 141].

#### Metabolism of ayahuasca alkaloids

The metabolic pathways for DMT and the *β*-carbolines in ayahuasca are well understood (Fig. 1). In additional to the extensive first pass metabolism or oral DMT, there is also rapid second pass oxidative deamination via MAO-A in brain [103] and other tissues, irrespective of the route of administration. After oxidative deamination, the second-most important metabolic route for DMT is to DMT-*N*-oxide (DMT-NO) via unspecified hepatic cytochrome P450 (CYP450) enzymes, with minor routes resulting in the production of *N*-methyltryptamine (NMT) or 6-hydroxy-DMT (Fig. 1). Of these metabolites, the former compound is anecdotally psychoactive, according to Shulgin [3]. Recent studies indicate that the CPY2D6 and CYP2C19 cytochrome oxidase isoforms can contribute to the formation of NMT from DMT [109, 110]. However, the specific isoform/s responsible for the conversion of DMT to 6-hydroxy-DMT remain unknown. Inhibition of MAO-A, by reducing or slowing the production of 3-IAA, shifts the branching ratio in favor of the secondary metabolic pathways. Thus, MAO inhibition augments the formation of DMT-NO, NMT, and 6-hydroxy-DMT [107]. In a rat study with DMT administration alone (1 mg/kg i.p.), the brain concentration of 3-IAA at 100 min was ~ 50-fold higher than that of unmetabolized DMT. However, with co-administration of harmine (1 mg/kg i.p.), the brain exogenous alkaloid concentrations were 34% DMT, 65% 3-IAA, and 1% DMT-NO [103]. Thus, even with substantial (but incomplete) MAO inhibition, 3-IAA remained the main metabolite in brain. In an analysis of 24-h urine samples collected after ayahuasca administration, there was 1% recovery of unchanged DMT, versus 55% as 3-IAA and 12% as DMT-NO [108], suggesting that DMT-NO formation may be more important systemically than in brain (DMT-NO is unlikely to cross the blood–brain-barrier). In another urine analysis study, there was 97% excretion of the DMT dose as 3-IAA and 3% as DMT-NO after oral administration [6]. In contrast, that study showed significantly higher generation of DMT-NO (28%) after smoking, with 63% excreted as 3-IAA and 10% leaving the body unchanged. Despite the lacking MAO-A inhibition in that study, renal elimination as DMT-NO exceeded that seen after ayahuasca administration.

Harmine and harmaline are metabolized in the body to hydroxy-harmine or and hydroxy-harmaline by enzymes from the CYP450 family, or to harmol and harmalol [107]. Similarly to harmine and harmaline, THH is preponderantly metabolized to tetrahydroharmol [107, 108], but the responsible enzymes remain to be established. In 24-h urine samples collected after ayahuasca administration, there were low total recoveries of harmine, THH, and their metabolites as compared to DMT and harmaline recovery, which comprised approximately two-thirds of the administered dose [108].

### Molecular and cellular mechanisms

#### Molecular targets of DMT and ayahuasca

Conventional understanding links the psychedelic properties of DMT (and ayahuasca) to agonism at brain serotonin 5-HT_2A_ receptors [52]. However, DMT has only modest affinity at these receptors in vitro (K_i_ = 127–1200 nM and IC_50_ = 75–360 nM) [142–146]. Additional binding at serotonin 5-HT_1A_ (K_i_ = 183 nM, IC_50_ = 170 nM) and 5-HT_2C_ receptors (K_i_ = 360–2630 nM, IC_50_ = 360 nM), along with other receptor subtypes, have been proposed to contribute to the overall psychoactive effects of DMT [142–150]. 5-HT_1A_ receptors predominantly occur in the limbic system and brain regions that receive projections from other parts of the limbic system, such as the amygdala, hippocampus, cingulate cortex, and certain other neocortex regions [151, 152]. In these regions, the 5-HT_1A_ receptors have post-synaptic localization, while 5-HT_1A_ receptors in the raphe nuclei are pharmacologically distinct autoreceptor sites that control serotonin release and firing rate [153]. The 5-HT_1A_ receptors are mechanistically relevant for the biological understanding of depression [151, 152], as 5-HT_1A_ agonism proposedly improves stress resilience [154], and modulates HPA axis functioning [155] and neuroplasticity [156]. Not only DMT, but also the ayahuasca *β-*carbolines influence serotonin neurotransmission, either directly (DMT as a 5-HT_1A/2A/2C_ agonist) or indirectly (THH as a SERT blocker and weak MAO-A inhibitor, and harmine and harmaline as potent MAO-A inhibitors), which could relate to reported anti-depressant effects of ayahuasca [157]. Interestingly, the co-administration of the 5-HT_1A/1B_ receptor partial agonist pindolol potentiated the subjective effects of DMT in a human trial [135], suggesting an autoreceptor regulation of the post-synaptic effects of DMT.

5-HT_2A_ receptors have highest expression in brain in layer 5 pyramidal neurons in the neocortex, but also occur in limbic and basal brain structures [154]. As noted above, DMT shows moderate affinity towards 5-HT_2A_ sites, as does harmine (K_i_ = 230 nM), whereas harmaline and THH show very low 5-HT_2A_ affinities of 7.8 and > 10 µM, respectively [158, 159]. Νotably, pre-administration of the serotonin 5-HT_2A/C_ blocker ketanserin (as tartrate, 40 mg) significantly diminished (but did not ablate) the neurophysiological and subjective effects of ayahuasca reported by participants via the hallucinogen rating scale (HRS) and the altered states of consciousness (ASC) questionnaire [141]. There were significant reductions in the HRS subscales affect, perception and intensity, and in the ASC subscale “visionary restructuralization” upon ketanserin pretreatment. However, these subscale scores were still significantly higher than on study days without ayahuasca administration, which implies that 5-HT_2A_ receptors may not be the solitary site of DMT action. There was no significant ex vivo occupancy by DMT plus harmine (1 mg/kg, each) at rat cortical 5-HT_2A_ receptors labelled with [^3^H]ketanserin [103], a close analogue of the PET ligand [^18^F]altanserin [160]). In the rat study higher doses of DMT plus harmine (3 mg/kg, each) also evoked no detectable occupancy at binding sites for [^18^F]MHMZ, a 5-HT_2A_ antagonist PET ligand with higher selectivity and binding signal than [^18^F]altanserin/[^3^H]ketanserin. Those negative results may call into question the contention that DMT acts exclusively via serotonin 5-HT_2A_ receptors. In another study, administration to rats of ayahuasca at doses containing 0.3 mg/kg DMT led to extinction of contextual freezing behavior [161]. With repeated ayahuasca doses, the co-administration of the 5-HT_2A_ receptor antagonist MDL-11,939 or the 5-HT_1A_ receptor antagonist WAY-100635 in the limbic cortex blocked the fear extinction effects, again suggesting an action at both receptor types [161]. The 5-HT_2C_ receptors have expression in epithelial cells in the choroid plexus and GABAergic neurons in prelimbic prefrontal cortex (PFC), and in other cortical, limbic, and basal ganglia regions, where they may present targets for various neuropsychiatric disorders [162]. DMT and harmine both show low affinity to 5-HT_2C_ receptors [158], but we cannot presently exclude an action of ayahuasca at these sites.

While LSD interacts at dopamine D_2/3_ receptors in vitro [143, 163] and in vivo [164], DMT has little affinity at dopamine receptors [128]. However, the indisputable involvement of brain dopamine in affective disorders, reward learning, and avoidance behaviors in relation to anhedonia [165, 166], we may infer an indirect action of ayahuasca at dopaminergic pathways. While ayahuasca *β*-carbolines likewise have little affinity for dopamine receptors [167], they may yet mediate indirect effects on brain dopamine via MAO-A inhibition [157]. Thus, for example, ayahuasca administration increased the dopamine concentration in amygdala of rats [168]. Nonetheless, as noted above, complete blockade of both forms of MAO did not potentiate the amphetamine-evoked dopamine release in the [^11^C]raclopride PET competition paradigm [169, 170]. On the other hand, local application of harmine (300 nM) substantially increased the electrically evoked release of dopamine in nucleus accumbens brain slices, in a manner seemingly unrelated to MAO inhibition, but apparently involving 5-HT_2A_ receptors [171]. Harmine may inhibit dopamine reuptake via DAT [107] and may somehow contribute to the normalization of aberrant DAT membrane trafficking and DA reuptake rate in addictive disorders [172, 173]. Sigma-1 receptors, which are abundant throughout the CNS [157, 174], are another potential site of DMT action. However, the reported affinities for DMT towards sigma-1 receptors (K_D_ = 14 µM [174], K_i_ = 5.2–15.1 µM [143, 175]) may not suffice to impart significant effects. Nonetheless, DMT induced reductions in electrophysiological measures (spreading depolarization), which were normalized by co-administration of sigma-1 antagonists NE-100 and asenapine [175]. The selective sigma-1 receptor agonist PRE-048 evoked a similar reduction in spreading depolarization. Additional immunohistochemistry results in the same study indicate that DMT might have neuroprotective properties against hypoxia or ischemic stroke [175].

The *β*-carbolines harmine and harmaline are antagonists at alpha-1 adrenergic receptors, with IC_50_ values in the range 31–36 µM [176], and may inhibit acetylcholinesterase, which would thereby potentiate cholinergic neurotransmission [157]. Other possible actions of harmine include modulation of GABAergic neuronal transmission [177] and inhibition of intracellular protein aggregation (perhaps relevant in neurodegeneration models) [178], which may call for further investigation of therapeutic mechanisms [157]. Harmine exerts anti-inflammatory, neuroprotective, antidiabetic, and antitumor effects in various models [179–182]. Overall, the ayahuasca *β*-carbolines may have effects extending beyond simple MAO-A inhibition, but with uncertain relevance to ayahuasca psychopharmacology.

#### Neuroplasticity induced by DMT and *β*-carbolines

Recent research addresses the possibility that psychedelic substances can induce or reinstate neuroplasticity, e.g., by altering gene and protein expression, post-translational processes, synapse formation, or neurogenesis. While most such studies have concerned psilocybin, there are a few reports on neuroplastic effects of DMT and the ayahuasca *β*-carbolines (for review, see [183]. Especially in human research, plasma levels of brain-derived neurotrophic factor (BDNF), a neurotrophin known to regulate synaptic plasticity and neuronal growth [184], have served as a marker for potential effects of neurogenesis in the context of antidepressant treatment [185]. While one study showing increased plasma BDNF levels after ayahuasca intake by healthy and depressed individuals [186], other studies with ayahuasca or DMT showed no significant changes [7, 187]. In a preclinical study, there was likewise no increase in plasma BDNF after DMT administration. However, co-treatment with an antagonist of tropomyosin receptor kinase B (TrkB, the high affinity receptor for BDNF), or with an inhibitor of downstream target of TrkB signaling (mTOR), completely blocked the neuroplastic effects of DMT, suggesting significant engagement of the BDNF signaling pathway in mediating neuroplasticity [188]. In that same study, a single treatment i.p. with DMT (10 mg/kg as free base) increased dendritic spine density and neuronal excitability in PFC neurons, which might explain the antidepressant and fear extinction effects reported in another rat study with DMT [189]. Increased dendritic spine growth was observed after activation of intracellular 5-HT_2A_ receptors with DMT, psilocin or psilocybin. These intracellular receptors are mostly inaccessible by endogenous serotonin, thus suggesting that DMT might induce neuroplasticity via an intracellular mechanism, possibly also at the low endogenous concentrations [190]. Chronic microdosing (0.77 mg/kg DMT freebase (as hemifumarate) 2–3 times per week for 7 weeks) did not alter BDNF levels or 5-HT_2A_ receptor expression in rats, but nonetheless exerted antidepressant-like behavioral effects and improved fear extinction learning without other seemingly negative behavioral changes [191]. Interestingly, the authors also reported retraction of dendritic spines in the PFC, but only in female DMT-treated rats. These latter effects may raise concern about the possibility of unfavorable effects with excessive or prolonged microdosing regimens [191]. Many of the presented findings potentially link to biomolecular underpinnings of affective disorders, e.g. decreased BDNF levels or TrkB signaling could underly depression, or neuroinflammation due to immunological hyperactivity could mediate anxiety symptomatology [25, 185, 192, 193]. DMT treatment enhanced performance in memory tests and spatial learning in adult mice, while promoting neurogenesis in the subgranular zone of the hippocampus in vitro (tested after 7 days) and in vivo (2 mg/kg repeated doses of DMT either daily over 4 days, or every other day for 21 days) [194]. Co-administration of a sigma-1 receptor antagonist blocked these effects, which may belie the low affinity reported for DMT at that binding site.

Preclinical studies have implicated harmine as an enhancer of BDNF signaling in rat hippocampus, in association with antidepressant-like effects in a behavioral assay, both for acute and chronic administrations [25, 193]. However, other rat studies showed that a high dose of harmine (15 mg/kg as harmine hydrochloride) induced anhedonia in the sucrose preference test, and reduced locomotor activity, without increasing hippocampal BDNF levels [195]. All three main *β*-carboline alkaloids in *B. caapi* promoted neurogenesis in an in vitro assay with progenitor cells from the subventricular and subgranular zone, which are the main niches of adult neurogenesis in mice. Harmine, harmaline and THH all significantly increased stem cell proliferation, migration, and eventual differentiation into neurons to assays in vitro [196]. Complementing these findings, earlier studies in chick embryo cells [197] and human neural progenitor cells [44] showed that harmine (2–5 µM in chick embryo and 7.5–22.5 µM in human progenitor cells) increased mitosis rates. In a mouse model of anxiety, harmine (20 mg/kg i.p. daily for 7 days) reduced anxiety-like behavioral effects and blunted neuroinflammation in the basolateral amygdala [192].

We emphasize that some studies have reported adverse effects from very high or repeated doses of DMT or ayahuasca [121, 191, 195], in keeping with Paracelsus’ dictum *dosis sola facit venenum* (only the dose makes the poison). As with any medication, exceeding some therapeutic dose range may offset beneficial effects of appropriate dosage regimens. The involvement of BDNF signaling in the effects of DMT/ayahuasca seem relevant to the association of BDNF with models of depression and anxiety disorders arising from a hyperactive immune system and chronic low-grade inflammation [25, 185, 192, 193]. As substantiated by the burgeoning publications on neuroplasticity in the psychedelics literature [183], there is growing interest in the basic biological mechanisms of action of psychedelic substances. A simple model in which DMT and other ayahuasca constituents act exclusively at serotonin 5-HT_2A_ receptors falls short of explaining the full spectrum of acute and chronic effects.

### Functional mechanisms—human brain imaging and EEG studies

We now give a narrative account of the available molecular imaging, fMRI, and EEG studies reporting effects of ayahuasca (14) or DMT (9) on human brain function. We present the studies in chronological order in Supplementary Table 1, including a brief description of the study design, sample, and interventions, along with key results, with a more detailed discussion in the following section.

#### Neuroimaging studies with ayahuasca and DMT

The first ayahuasca neuroimaging study used single photon emission tomography (SPECT) to determine the acute effects of lyophilized ayahuasca capsules on regional cerebral blood flow (CBF) [198], a surrogate marker for neuronal network activation. Ayahuasca administration increased perfusion in the right hemisphere anterior cingulate cortex (ACC) and medial frontal gyrus, bilaterally in the anterior insula and inferior frontal gyrus, and in the left amygdala and parahippocampal gyrus. These regions are thought to play key roles in interoception, body awareness, and emotional processing [199, 200], well aligning with the acute subjective effects of ayahuasca [46, 201]. A similar SPECT study in depressed patients showed significantly increased perfusion in the left nucleus accumbens (NAc), right insula and left subgenual area 8 h after ayahuasca treatment compared to baseline [29]. Additionally, acute reductions in depressive symptoms (80–180 min after administration) persisted up to three weeks. Previous neuroimaging studies (deep brain stimulation, PET, fMRI) have shown hypoactivity in precisely these regions in depressed patients, which rectified upon treatment with conventional antidepressants such as SSRIs or deep brain stimulation [202–206]. Post-acute results from the depressed group showed only partial overlap (in the right insula) with the acute effects of ayahuasca on cerebral perfusion in the healthy volunteer study [29, 198], which might reflect changes in neuronal responsivity to the pharmacological challenge or time-dependent measurement differences.

In two task-based fMRI studies during acute DMT (i.v.), the first study showed no significant changes in blood oxygenation level dependent (BOLD) signal, despite the participants’ reduced reaction time to stimuli [207], whereas the second study showed signal reductions in brain regions associated with processing visual and auditory information in addition to reduced reaction time [208]. These combined behavioral and fMRI results recapitulated earlier behavioral findings with two different DMT doses [209]. Participants in another study with somewhat higher doses of i.v. DMT reported experiencing pronounced elementary and complex imagery [8], which might explain the reduced capability to focus on such attention-based tasks.

Another fMRI study investigating mental imagery during acute ayahuasca effects reported increased BOLD signal in many brain regions compared to baseline, including bilateral cuneus and left precuneus, lingual gyrus, fusiform, parahippocampal and temporal, occipital and frontal gyri [210]. These changes occurred during an imagery experience and may underly the often-reported vivid internal visual alterations with closed eyes. Partially overlapping results were reported in [198], and correspond to functional representations such as the peripheral visual field, retrieval of episodic memories, processing of contextual associations, and mental imagery. Changes in functional connectivity during mental imagery after ayahuasca intake, indicate alternations in the top down temporal information flow between frontal and occipital regions. Visions produced by ayahuasca seemingly arise in the primary visual cortex (V1) [210] and propagate to higher order visual regions. Another report of the same study sample showed changes in the default mode network (DMN) with task-based (verbal fluency) and with resting-state (rs) fMRI recordings [43]. Six of the nine pre-defined DMN regions showed significant activity decreases when comparing rest to task periods and ayahuasca to baseline. Two of the remaining DMN ROIs (left MFG and left MTG, involved in language processing [211]), also showed significant BOLD signal decreases. Additionally, functional connectivity declined within PCC/precuneus after ayahuasca intake. These findings suggest that experienced ayahuasca users achieve a brain state that occurs with decreased mind-wandering, allowing them to observe their thoughts and feelings without judgment, similar to experienced meditators [212].

In a follow-up analysis of the same rs-fMRI data increases of global entropy (Shannon entropy, expressing the uncertainty or variability in stochastic variables) were identified. Increases of local integration and decreases of global integration in various brain networks [213] imply that ayahuasca altered the modular structures of resting state networks. These results align with the *entropic brain hypothesis*, which proposes that psychedelic states entail higher entropy than ordinary waking consciousness [214].

A proton magnetic resonance spectroscopy ([^1^H]-MRS) and rs-fMRI study with baseline and post-acute measurements one day after ayahuasca ingestion showed decreased glutamate + glutamine, creatinine + phosphocreatinine, and *N*-acetylaspartate + *N*-acetylaspartylglutamate signals in the PCC [215]. These lower metabolite levels indicate higher neuronal activity during acute ayahuasca intake [215]. Indeed, other psychedelics evoked decreased inhibitory alpha-waves in similar brain regions as in ayahuasca studies [141, 216, 217], and similar MRS changes occur in in patients successfully treated for depression with cognitive behavioral therapy (CBT) or SSRIs [218]. Complementary rs-fMRI measurements revealed enhanced crosstalk between the ACC (associated with executive and cognitive-emotional processing) and the PCC and limbic structures (highly relevant for emotion and memory processing), which may relate to the antidepressant effects of ayahuasca [215]. Parts of the salience (SAL; ACC) and the DMN (PCC) networks are habitually anti-correlated in normal waking consciousness [219], but enhanced coupling may occur during ayahuasca and other psychedelic experiences [220]. A later study replicated those findings of post-acute increased connectivity between the SAL and the DMN one day after administration of ayahuasca to healthy volunteers [221]. In accordance with previous reports, increased ACC connectivity within the salience network and decreased connectivity in the PCC within the DMN were identified [221]. A novel approach was adopted in a rs-fMRI study in a group setting with members of the Santo Daime church presenting “connectome fingerprints” for each participant, based on the idea that functional connectivity is more consistent within the same person across repeated scans than between different subjects [222]. Participants showed greater alignment between connectomes during the acute ayahuasca phase than during the placebo scans. After ayahuasca treatment, network stability decreased in the SAL, and increased in the dorsal attention network (DAN). Between-network stability mostly decreased from the SAL and visual network (VIS), extending to the other five large-scale brain networks defined by Yeo and colleagues [223].

Similarly, i.v. DMT administration decreased within-network integrity in five (VIS, somatomotor network (SM), DAN, fronto-parietal network (FP) and DMN) of the seven Yeo networks, and increased within-network functional connectivity in the SAL, FP and DMN [224]. An increased between-network functional connectivity between the FP, DMN, and SAL networks, and the other Yeo networks, suggest that DMT mainly affects networks integrating and processing higher cognitive functions. Additionally, DMT flattened the principal cortical gradient acutely [224] (which ranges from areas for processing sensory and motor information to regions subserving higher cognitive processing [225]). These findings suggest that DMT transiently dysregulates functional hierarchies, enabling greater cross-communication between brain regions and networks as compared to ordinary consciousness.

Another task-based fMRI study testing implicit aversive stimulation showed longer reaction times to aversive compared to neutral images at baseline, whereas during the ayahuasca scan reaction times did not differ according to emotional valence of the visual stimulus [226]. Furthermore, in the ayahuasca condition, there was decreased activation of the bilateral amygdala and increased activation of bilateral insula and right dorsolateral PFC upon exposure to aversive images. These findings align with previous ayahuasca studies, showing activation of regions involved in emotion processing, such as the amygdala, ACC, and insula, possibly related to the intensified emotional experience [29, 198].

#### EEG studies with ayahuasca and DMT

The first open-label EEG study during the ayahuasca ritual among healthy members of the Santo Daime church reported increases in gamma power in the left posterior temporal cortex and the left occipital lobe with close eyes and increased gamma power in the central, parietal, and occipital lobes with open eyes compared to baseline [227]. Gamma is a high frequency (30–80 Hz) band modulated by external sensory inputs and internal processes such as working memory and attention; as such, their EEG findings bear some relation to fMRI findings of increased activity (e.g., in visual cortex, fusiform gyrus or prefrontal cortex) during a mental imagery task after ayahuasca administration [210]. A later study found a dose-dependent reduction in EEG power across all frequency bands, peaking between 90 and 120 min after ayahuasca administration [228]. Additional EEG analyses focussing on peak effects at 60 and 90 min after high doses [229] showed widespread bilateral decreases in alpha and delta power in somatosensory, auditory, and visual association cortices. At 90 min even greater reductions in beta, delta, and theta power occurred in cortical regions relevant for emotion and memory processing. Similar decreases in lower frequency (delta and theta) power have been observed after treatment with other psychedelics, or psychostimulants [230, 231].

Delta waves usually increase during deep sleep, or in meditative and relaxed states, so decreased delta power might suggest an excitatory effect of ayahuasca [229]. This would be in accordance with animal studies showing excitatory postsynaptic potential and currents after psychedelics administration [232]. Although delta and theta power in EEG recordings was unchanged in a later study up to two hours after ayahuasca intake [137], alpha power declined in parieto-occipital regions at 50 min. Some cortical regions showed increased slow-gamma power between 75 and 125 min, while fast-gamma power had decreased in four clusters during the same time window. DMT and *β*-carboline plasma levels showed positive correlations with EEG power in the beta, gamma and delta bands, and a negative correlation with alpha power. Specifically, DMT and harmine levels correlated more strongly with early phase alpha power decreases, while the harmaline and THH concentrations correlated more strongly with the late phase gamma band increases. These changes in *β*-carboline profiles matched earlier pharmacokinetics findings [10, 11].

In an EEG study using transfer entropy (TE) to measure directed information transfer ayahuasca administration resulted in decreased information flow from frontal to posterior brain regions and increased flow from posterior to frontal regions [233]. These changes, observed at various time points after administration, suggest that ayahuasca disrupts the usual neural hierarchies between higher order frontal regions and more sensory-related posterior regions, aligning with similar findings from fMRI studies with i.v. DMT [224].

An EEG study that tested the effects of ayahuasca and ketanserin in a 2 × 2 design found decreases in alpha, delta, and theta frequency bands after 90 min [141], much as in the above-mentioned earlier studies [137, 228, 229]. Ketanserin alone had opposite effects, with unchanged alpha power but increased delta and theta powers compared to placebo. When combined, ketanserin and ayahuasca led to stronger increases in delta and theta power, counteracting ayahuasca’s effects. Ketanserin before ayahuasca reduced, but did not completely block all subjective effects, possibly related to changes in EEG bands.

Performance of a cognitive mismatch negativity (MMN, a brain response to violations of a rule) EEG task decreased dose-dependently during i.v. DMT administrations compared to baseline [234]. After the low DMT dose, there was diminished N1 peak amplitude (~ 150 ms after stimulus), indicating decreased attention to visual stimuli [235], and attenuated MMN signal in the right hemisphere. Similarly, psilocybin treatment also reduced N1 peak activity, albeit with stronger effects on MMN [236, 237].

A more recent EEG study with i.v. DMT showed significantly increased signal diversity and delta and gamma powers, while decreasing alpha and posterior beta powers, correlating with intensity ratings and DMT plasma levels [238]. A cortical travelling wave analysis revealed increased forward waves (FW) and decreased backward waves (BW) [239]. After DMT administration, the frequency of the travelling waves decreased for alpha and beta and increased for delta and theta, matching previously reported frequency band power changes. These changes resembled those seen during visual stimulation [240], suggesting a mechanism for DMT-induced visual hallucinations [8, 241]. Another analysis modelled the relationship between alpha and beta power, signal complexity and simulated DMT plasma levels, identifying specific concentrations evoking half-maximal (IC_50_) band reductions in alpha (71 nM) and beta power (137 nM), and the EC_50_ for signal complexity (54 nM). These results constitute the first dose–response relationship for EEG signal strength with DMT.

EEG recordings after self-administration of DMT by smoking in a naturalistic setting recapitulated findings [238] of a widespread reduction in alpha power lasting several minutes, along with reduced delta and gamma power in occipital, parietal, temporal and antero-central regions during the same time window [242]. In a separate reanalysis of these data, the same pattern of power changes was found alongside a negative correlation with subjective effects only with theta power changes [243].

An additional analysis of the study presented above [224] focused on the relationships between EEG and simultaneous fMRI findings after i.v. DMT administration. EEG data showed reduced alpha and beta power, decreased fractal spectral power below 30 Hz, and increased signal complexity. Significant negative correlations were found between intensity ratings and plasma DMT concentrations with alpha- and beta-power and positive correlations with delta- and theta-power, much as reported in [238]. The cross-model analysis showed positive correlations between frontal delta power and negative correlations between parietal alpha-power with GFC in most RSNs, along with positive correlations between gamma power and signal diversity in a few RSNs. This study reinforced preceding EEG findings with additional fMRI data, demonstrating the benefits of multimodal neuroimaging.

In reviewing EEG studies on ayahuasca and DMT, two main findings consistently appear: (1) a reduction in alpha frequency power, and (2) an increase in signal complexity. These effects occur 60–120 min after ayahuasca administration and shortly after i.v. or inhaled DMT [137, 141, 224, 228, 229, 238, 242, 243]. Reduced alpha power has been linked to increased brain metabolism [244–246], which could explain the increased BOLD signal in the visual cortex [210], and the vivid visual effects often reported with these substances. The alpha band is the most prominent feature of resting-state EEG recordings in adults [247], and is linked to high-level psychological functioning [248, 249] and top-down brain regulation [250, 251], both of which are generally modified by psychedelics [224, 233, 252, 253]. While changes in other frequency bands are less consistent, the increase in signal complexity supports the idea of decreased top-down regulation and hierarchical processing of neural information during the psychedelic state [214, 252].

Neuroimaging and EEG studies on DMT and ayahuasca vary widely in their designs, dosages, administration routes, and settings, which can make comparisons difficult. However, many studies focused on resting-state EEG or fMRI, which are useful for examining brain activity without specific tasks and can be easily compared. Despite these caveats, these studies have informed several models of psychedelic action emerging over the past few years. These models include the entropic brain hypotheses as noted above [214] and its generalization, the REBUS (relaxed beliefs under psychedelics and the anarchic brain) model [252], the cortico-striato-thalamocortical (CSTC) model [254], the strong priors (SP) model [255], and the cortico-claustro-cortical (CCC) model [256]. The CSTC and the CCC model mainly rely on the assumption that 5-HT_2A_ receptor agonism is the key driver for psychedelic effects, which is less than certain for the case of DMT/harmine. The REBUS model proposes that psychedelics reduce the influence of top-down processes (like expectations or prior beliefs) and thereby enhance bottom-up sensory and emotional information flow potentially facilitating therapeutic change processes. We prefer the REBUS model because it explains several observed effects such as the reduction of a cortical gradient (fMRI), modulations of RSNs (especially the DMN), increased signal complexity (EEG) or altered direction of cortical traveling waves in the alpha band [257].

For a comprehensive integration of these models into general neuroscientific research on psychedelics, see [258]. Several systematic reviews have examined neuroimaging studies with various psychedelic substances, e.g., Gattuso et al. [259] and McCulloch et al. [260]. These reviews highlight the modulation of the DMN and resting-state brain activity under the influence of psychedelics, using various imaging techniques (e.g. fMRI, EGG, MEG). However, there is a notable lack of molecular imaging studies specifically examining the actions of DMT and ayahuasca.

### Long-term psychological and neuroanatomical changes after ayahuasca use

In a cross-sectional study, Bouso et al. [261] compared long-term ayahuasca users (n = 127) of various ayahuasca churches in Brazil with a religious control group (n = 115) that abstains from substance consumption. Psychometric assessments were conducted at study inclusion (T1) and one year later (T2). The ayahuasca group showed significantly lower Harm Avoidance and Self-Directedness, higher Self-Transcendence, and lower general subjective symptoms, which suggests that consumption in religious contexts is beneficial for mental and physical health. Additionally, ayahuasca users scored higher on all subscales of the Spiritual Orientation Inventory [262] at both time-points, indicating a reinforcement of spiritual beliefs and attitudes. They also performed better in neuropsychological tests of conflict monitoring, executive function, and working memory at both time-points.

Structural MRI provides quantitative measures of brain volume and cortical thickness, which can indicate structural changes due to aging, neurotoxicity, or neuroplasticity. Bouso et al. [263] reported on cortical thickness in experienced ayahuasca users from the Santo Daime with a matched control group. The habitual ayahuasca users showed significant cortical thinning in middle and inferior frontal gyrus, precuneus, superior frontal gyrus, PCC, and superior occipital gyrus, along with cortical thickening in the precentral gyrus and ACC. Cortical thinning in the PCC negatively correlated with lifetime and years of ayahuasca use. Neuropsychological tests revealed the same differences as the cross-sectional study mentioned above. The cortical thinning in key nodes of the DMN might imply long-term downregulation of DMN activity, although no direct evidence links this to reduced within-DMN connectivity [43, 224]. The negative correlation of PCC thickness and self-transcendence might imply an anatomical basis for the increased religiosity often reported by ayahuasca users. Overall, Bouso’s studies suggest that regular, long-term ayahuasca use in religious contexts benefits brain health, though these findings may not necessarily apply to a secular or psychiatric use. The general abstinence from other drugs among ayahuasca church members may support the cognitive benefits attributed to ritual use.

Another analysis of the same MRI study tested the hypothesis that regular ayahuasca use would lead to thickening of fiber tracts in the corpus callosum, the bundle connecting the cerebral hemispheres [264]. Results showed thickening in the isthmus of the corpus callosum, correlating with ayahuasca use frequency, although these findings did not hold after multiple testing corrections. The implicated callosal regions are positioned to mediate increased interhemispheric connectivity between motor and somatosensory regions [265]. Obtaining such an effect might be beneficial for motor function, various neurodegenerative disorders, e.g. amyotrophic lateral sclerosis (ALS), and for stroke rehabilitation [265, 266].

A more recent cross-sectional structural MRI study assessed morphometric similarity (MS), an approach whereby anatomical connectivity is analyzed by considering multiple brain structural features (e.g. grey matter volume, and cortical curvature and thickness) [267], in 24 frequent ayahuasca users from the Santo Daime church and 24 matched controls [268]. The ayahuasca group showed overall reduced MS compared to controls, with lower MS in sensorimotor cortices (inferior frontal gyrus, precuneus, pre and post central gyrus) and higher MS in the orbitofrontal, entorhinal, cingulate, and anterior insular cortices. Lower MS indicates *decreased* anatomical connectivity between a region and the rest of the cortex [268]. The findings of increased MS in the ACC align with an earlier MRI study showing thinning [263]. MS analysis with the seven RSNs defined by Yeo and colleagues [223] revealed reduced MS in the sensorimotor, dorsal attention, and default mode networks for the ayahuasca group, with increased MS in the limbic network. Additional correlations with gene expression maps in the ayahuasca group revealed 18 genes relevant to DMT or ayahuasca effects (with either positive or negative weightings) on MS findings. Notably, 5-HT_2A_ receptor gene downregulation in sensorimotor cortices correlated with lower MS, suggesting that repeated ayahuasca use may lead to sustained downregulation and desensitization, similar to LSD tolerance [269]. Since ayahuasca or DMT do not seem to evoke rapid tolerance [62], it is questionable if such 5-HT_2A_ receptor downregulation could mediate tolerance. Earlier neuropsychology studies showed that regular ayahuasca users had better integrity of executive functions than less experienced users [270], which might relate to 5-HT_2A_ receptor desensitization or downregulation, a matter for future PET studies.

Pharmacological models alone may not fully explain the anatomic/functional associations observed in ayahuasca studies. For example, religiosity in Christian church members showed associations with a widespread pattern of greater cortical thickness [271], compared to the more diverse findings of cortical thickness changes in ayahuasca users. The cross-sectional studies of Santo Daime church members, who abstain from other drugs, provide a unique opportunity to study repeated ayahuasca use. However, these cross-sectional studies cannot infer causality. Larger, longitudinal studies are needed to confirm the structural and functional brain changes linked to ayahuasca use.

## Conclusions and future directions

With this narrative review and synthesis, we summarize the current state of research with DMT, *β*-carbolines, and ayahuasca. While DMT is the main psychedelic constituent, the diverse *β*-carboline alkaloids in ayahuasca contribute to its unique (adverse) effects, creating an entourage effect that distinguishes it from synthetic formulations. The combination of DMT with MAO inhibitors enhances its bioavailability and duration and offers alternatives to inhaled or injectable routes of administration, which lead to short but intense DMT experiences. We emphasize the need to distinguish between a reductionist view of ayahuasca as a mixture of chemical substances and its full context as a cultural practice, often affiliated with traditional and syncretic religions. The findings presented herein mostly fall within the broad field of neurobiology, and do not adequately accommodate the cultural and botanical knowledge associated with indigenous usage of ayahuasca for therapeutic and ritualistic purposes.

Current understanding does not definitively implicate a singular molecular target that could explain the subjective effects of DMT or ayahuasca. Key candidates include several serotonin receptor subtypes (5-HT_1A/2A/2C_ and potentially others), an indirect influence on dopamine transmission due to *β-*carbolines, and potential roles of TrkB and sigma-1 receptors. Better qualifying the importance of 5-HT_2A_ receptor activation by DMT (alone or in combination with MAOIs) shall call for dedicated pre-clinical and clinical molecular imaging studies.

Neuroimaging and EEG studies have consistently shown reductions in alpha frequency power and increased signal complexity after ayahuasca or DMT administration. These findings are in line with brain models of decreased top–down regulation and enhanced neural communication during the psychedelic state. Functional neuroimaging studies have revealed changes in brain network dynamics, such as increased connectivity between the salience and default mode networks, and activations of brain areas associated with processing of emotions and autobiographical memories. Understanding these mechanisms is crucial for developing targeted therapeutic applications. The preponderance of imaging studies and clinical studies have been exploratory with open-label designs. There is a pressing need for larger-scale, controlled clinical trials to establish the dose-dependence and persistence of long-term therapeutic benefits and neurobiological effects induced by ayahuasca or DMT. Such studies should integrate clinical scoring with advanced imaging methods. We also see a need for comparative studies with other classical psychedelics, aiming to understand better the neurobiological basis of their differing phenomenology. Promising findings that DMT and ayahuasca evoke neuroplastic effects call for consolidation with comparable results seen with other psychedelic compounds. The bulk of such research has hitherto entailed studies in vitro and in preclinical research, without translation to the human clinical context. Multimodal neuroimaging techniques such as diffusion tensor imaging (DTI) could serve to investigate changes in white matter tracts in conjunction with MRS to follow changes in specific neurometabolites acutely or over time [272]. Recent advances in neuroimaging data processing, e.g. the development of a modality-overarching neuroimaging data structure [273], standardization of fMRI preprocessing pipelines [274], and the development of sophisticated computational approaches should enable streamlining and standardization of neuroimaging results to facilitate easier interpretation between studies.

Additionally, molecular imaging studies, including PET, are essential to further explore receptor occupancy and neuroplasticity mechanisms in vivo. Neuroplasticity in response to ayahuasca or its constituents might be amenable to investigation by PET with a ligand for synaptic vesicle protein 2A (SV2A), as shown autoradiographically for the case of psilocybin in experimental animals [275]. PET studies with the serotonin 5-HT_2A/2C_ receptor agonist radioligand [^11^C]Cimbi-36 [276] (i.e., the psychedelic phenylethylamine generally known as 25B-NBOMe [277]) may be better suited than antagonist radioligands for establishing competition from the exogenous agonist DMT in vivo, as is the case for dopamine D_2/3_ receptors [278]. Similarly, an agonist PET ligand for 5-HT_1A_ receptors might serve to detect occupancy by DMT at this receptor in human brain. As noted above, the complex alkaloid composition of ayahuasca calls for consideration of the entourage effect, a concept originally known from cannabis research [67], and indeed in consideration of the polypharmacology of tobacco smoke, which evokes inhibition of MAO-A in human organs to [^11^C]clorgyline PET [279]. There were no effects in a pilot study of low dose harmine/DMT on energy metabolism in rat brain to [^18^F]FDG PET [103], but we are currently undertaking human [^18^F]FDG PET studies of pharmahuasca. Results of this ongoing neuroenergetics study could prove informative regarding the interpretation of the extensive fMRI/EEG literature on ayahuasca reviewed above.

In conclusion, the therapeutic potential of ayahuasca constituents to promote neuroplasticity and treat neuropsychiatric disorders, including depression, addiction, and PTSD, is gaining empirical support. Additionally, early evidence suggests a potential role of DMT in the treatment of acute brain injury such as ischemic stroke, which opens promising paths for future pharmacotherapeutic developments. Standardized formulations of DMT and harmala alkaloids present certain advantages for clinical investigations and basic research into the molecular pathways and mechanisms of action by offering more predictable pharmacokinetic and pharmacodynamic profiles, as well as better control of potential adverse effects, compared with botanical ayahuasca preparations. In addition to the molecular and neurophysiological perspective, we note the importance of processes of psychological change, alongside clinical and contextual factors such as supportive set and setting. While extending beyond this review’s original scope, we hold that such considerations are essential for obtaining a comprehensive understanding of the therapeutic potential of ayahuasca.

## Supplementary Information

Below is the link to the electronic supplementary material.

Supplementary file1 (PDF 243 KB)

## Data Availability

Not applicable.

## Footnotes

1 DMT is also present in a hallucinogenic snuff (known as yopo) derived from seeds of the South American trees *Anadenanthera peregrina* or *A. columbina*, along with the psychoactive tryptamines 5-methoxy-DMT (5-MeO-DMT) and 5-hydroxy-DMT (bufotenin) [280].
