# Supplementary material for: Neurobiological research on N,N-dimethyltryptamine (DMT) and its potentiation by monoamine oxidase (MAO) inhibition: from ayahuasca to synthetic combinations of DMT and MAO inhibitors
Source: Cell Mol Life Sci. 2024 Sep 10;81(1):395. doi: 10.1007/s00018-024-05353-6 (PMC11387584; doi:10.1007/s00018-024-05353-6)
Supplement: Supplementary file 1 — Supplementary file1 (PDF 243 KB) [file 18_2024_5353_MOESM1_ESM.pdf]

Supplementary Material for:

Neurobiological Research on *N,N*-Dimethyltryptamine (DMT) and its Potentiation by Monoamine Oxidase (MAO) Inhibition — From Ayahuasca to Synthetic Combinations of DMT and MAO Inhibitors

Klemens Egger[1,2,3*], Helena D. Aicher[1,2,4], Paul Cumming[3,5], Milan Scheidegger[1,2]

[1] Department of Adult Psychiatry and Psychotherapy, Psychiatric University Clinic Zurich and University of Zurich, Zurich, Switzerland
[2] Neuroscience Center Zurich, University of Zurich and Swiss Federal Institute of Technology Zurich, Zurich, Switzerland
[3] Department of Nuclear Medicine, Bern University Hospital, Bern, Switzerland
[4] Department of Psychology, University of Zurich, Zurich, Switzerland
[5] School of Psychology and Counselling, Queensland University of Technology, Brisbane, Australia

ORCID:
KE: 0000-0001-5072-9674
HDA: 0000-0001-5915-7086
PC: 0000-0002-0257-9621
MS: 0000-0003-1313-2208

*** Correspondence:**
Klemens Egger
Email: klemens.egger@bli.uzh.ch

Suppl. Table 1. Brain imaging and EEG studies with DMT or Ayahuasca.

	Study design	Study Sample	Study drug	Neuroimaging method	Key results
**Ayahuasca**					
Don et al. 1998 [1]	Open-label EEG study without a control group in a natural Santo Daime ceremony setting. EEGs were measured during Santo Daime ceremonies, 100 seconds of eyes-closed and eyes-open recordings were collected at baseline, then joined the ritual and 45–60 min returned to the test room to record again 100 sec of eyes-open and eyes-closed EEG.	11 healthy members of the Santo Daime church (5 female, mean age 36 years) with prior ayahuasca experience.	75 mL of ayahuasca was administered at every ceremony with a resulting mean dose of 41.3 mg DMT, 55.6 mg harmine, 4.6 mg harmaline, and 43.9 mg THH.	EEG; closed eyes and open eyes recordings	In the eyes-closed condition, effects of ayahuasca showed an increase in gamma power at 36–44 Hz in the left posterior temporal cortex and in the left occipital lobe ($p < 0.05$). For the eyes-open condition, gamma power was increased in the central, parietal, occipital cortex and in the right posterior temporal lobe ($p < .05$).
Riba et al. 2002 [2]	Double-blind, placebo-controlled, randomized clinical trial with 4 experimental sessions. Session 1 was single-blind placebo administration, sessions 2 to 4 were double-blind and consisted of one low dose, one high dose, and a randomly selected repetition of either low or high dose or placebo. EEG recordings were collected at baseline and in regular intervals after drug administration on each session, i.e. 15 min before, at timepoint of drug administration, 15, 30, 45, 60, 90, 120, 150, 180, 210, 240, 360, and 480 min after drug administration for 3 min for every recording.	18 healthy subjects (3 female, mean age 26 years) with at least 5 prior experiences with psychedelic substances.	Lyophilized ayahuasca capsules in two doses: low (0.5 mg/kg DMT, 1.02 mg/kg harmine, 0.07 mg/mL harmaline and 0.82 mg/kg THH) and high (0.85 mg/kg DMT, 1.44 mg/kg harmine, 0.10 mg/kg harmaline and 1.15 mg/kg THH).	EEG; closed eyes vigilance controlled	Subjective effects assessed with the Hallucinogen Rating Scale (HRS) showed significant dose-dependent increases in all subscales. EEG showed significant changes in the low dose group between 45 min and 2.5 hours after administration and more pronounced effects of the high dose from 1 hour to approx. 6 hours after administration with peak changes between 1.5 and 2 hours after administration compared to placebo. The total power was reduced in a dose-dependent manner after ayahuasca administration, while the centroid of total activity and standard deviation were increased significantly with peak increases at 120 and 90 after administration. Alpha, beta, delta, and theta bands analyzed individually all showed dose-dependent decreases in activity with temporal peaks between 90 and 120 min after administration.
Riba et al. 2004 [3]	Same study design as in Riba et al. 2002, but only one placebo session and the high dose condition were considered for analysis in this publication.	Same sample as in Riba et al. 2002.	Same dose as in Riba et al. 2002, but only the high dose data was used in this study.	EEG; closed eyes vigilance controlled	Omnibus significance test showed highest number of suprathreshold voxels at 60 and 90 min after ayahuasca administration, thus only these two timepoints were considered for consecutive analysis. Significant decreases ($p < 0.05$, Holmes-corrected) in the alpha-2 frequency were found 60 min after ayahuasca administration in parietal, occipital, temporal and limbic lobes bilaterally, additional decreases in delta power was found in a few voxels at the border between left occipital and temporal lobes. The highest number of significant voxels was found after 90 min, with significant decreases in delta (in parietal, occipital, temporal and limbic lobes), theta (frontal, temporal and limbic lobe) and beta (parietal, occipital, temporal and limbic lobe) power, alpha power reductions did not reach significance anymore.
Riba et al. 2006 [4]	Randomized, double-blind, placebo-controlled, within-subject. Ayahuasca capsules were administered 100–110 min before intravenous injection of the 99mTc-labelled ethylcysteinate dimer, with single frame imaging acquisition beginning one hour after tracer injection.	15 healthy male subjects (mean age 28 years).	Lyophilized ayahuasca capsules (1.0 mg/kg DMT, 1.7 mg/kg harmine, 0.16 mg/kg harmaline and 1.36 mg/kg THH).	[^{99m}Tc]-ECD SPECT	The administered dose reportedly elicited full-blown psychotropic effects. Significantly ($p < .002$, uncorrected) increased cerebral blood perfusion in the ACC and medial frontal gyrus in the right hemisphere, bilaterally in the anterior insula and inferior frontal gyrus, in the left hemisphere the amygdala and parahippocampal gyrus showed increased blood flow, no decreases in bloodflow in any brain region.
Alonso et al. 2015 [5]	Double-blind, randomized, cross-over study with 2 experimental sessions 1 week apart. In one session participants received placebo, in the other ayahuasca. EEG recordings were obtained	10 healthy male participants with previous psychedelics experiences.	Lyophilized ayahuasca capsules (0.75 mg/kg DMT, 1.28 mg/kg harmine, 0.12 mg/kg harmaline and 1.02 mg/kg THH).	EEG; closed eyes	Functional connectivity changes after ayahuasca administration were calculated with transfer entropy (TE). Ayahuasca induced significant TE changes 90, 120 and 150 min after administration, showing decreases at 90 and 150 min, and increases at the 120 min mark. After 120 min, the TE increases were directed by information from posterior regions and thus decreased uncertainty in

	at baseline, and at 15, 30, 45, 60, 90, 120, 150, 180 and 240 min after administration for 3 min each.				frontal regions. At 90 and 150 min after administration, the decreases in TE were directed by reduced influence of information in frontal regions directed to more posterior regions.
de Araujo et al. 2012 [6]	Within-subject fMRI study with 2 sessions per subject: one before ayahuasca intake (baseline) and one 40 min after ayahuasca intake on the same day. The task consisted of 3 conditions (natural image viewing, imagery with closed eyes, and scrambled image viewing) in a block design.	9 frequent ayahuasca users (5 female, mean age 29 years).	2.2 mL/kg ayahuasca (120-200 mL); the ayahuasca batch contained 0.8 mg/mL DMT, 0.21 mg/mL harmine.	fMRI; mental imagery task	Increased BOLD signal ($p < .05$, FDR-corrected) in various brain regions during mental imagery under the influence of ayahuasca, including the bilateral cuneus, left precuneus, lingual gyrus, fusiform, parahippocampal and temporal gyri, as well as occipital and frontal gyri. The authors observed dominant signal increases in the occipital cortex during natural image blocks. Comparisons of BOLD signal changes between task condition and scan revealed similarities between scrambled and natural image conditions both before and after ayahuasca intake, while the imagery condition showed a substantial increase in BOLD signal. The activated regions were associated with peripheral visual field, retrieval of episodic memories, contextual associations, and mental imagery. When analyzing functional connectivity changes during the imagery condition, connectivity patterns in primary visual cortex (V1) seemed to form a superposition of connectivity during baseline natural image and imagery conditions. There were also pattern changes (leading or lagging behind other BA areas) in BA10 (frontopolar prefrontal cortex) and BA19 (part of the extrastriate visual cortex) during the imagery condition after ayahuasca intake
Palhano-Fontes et al. 2015 [7]	Same study design as in de Araujo et al. 2021. Participants additionally performed a verbal fluency task for DMN activity analysis and additional resting state scan was used for DMN connectivity analyses.	Same sample as in de Araujo et al. 2012.	Same dose as in de Araujo et al. 2012.	fMRI; verbal fluency task and resting state	In the analysis of default mode network (DMN) nodes, selected based on a control group's verbal fluency task, six out of nine regions (ACC, PCC, precuneus, mPFC, MFG, left and right MTG and left and right IPL) showed a significant ($p < .001$) BOLD signal decrease during rest compared to task periods after ayahuasca intake. Functional connectivity analyses revealed a reduction in connectivity within the posterior cingulate cortex (PCC) and precuneus after ayahuasca intake, with no significant changes observed in the orthogonality between the DMN and task-positive network (TPN), more widely known as SAL.
Bouso et al. 2015 [8]	Retrospective study with control group.	22 regular ayahuasca users (16 female, mean age 41 years) from the Santo Daime church with at least 50 experiences in the two years before study participation and 22 matched controls (16 female, mean age 42 years).	No dose was administered within the study.	structural MRI	Anatomical changes ($p < .002$, cluster-size > 20): cortical thinning in middle and inferior frontal gyrus, precuneus superior frontal gyrus, PCC and occipital gyrus and cortical thickening in the precentral gyrus and ACC. The cortical thinning in the PCC showed a negative correlation with lifetime use of ayahuasca. Regular ayahuasca users showed lower harm avoidance ($p < .05$) and higher self-transcendence ($p < .001$) scores and performed overall better in 3 neuropsychological tasks targeting working memory, executive function and set-shifting ($p < .05$) than the control group.
Schenberg et al. 2015 [9]	Open-label EEG study without control group. EEG was recorded for 2 hours and divided into 10-minute segments that were analyzed together. The segments ended at 0 (baseline), 25, 50, 75, 100, 125 min after ayahuasca intake.	20 healthy subjects (8 female, mean age 29 years) with previous ayahuasca experience in non-religious contexts.	Administered ayahuasca was dose with 1.39 mg/kg DMT, 4.58 mg/kg harmine, 0.75 mg/kg harmaline, and 5.43 mg/kg THH.	EEG; resting-state with closed eyes	The EEG recordings did not show significant changes in the delta and theta bands after ayahuasca intake. Alpha power decreased 50 min after ayahuasca administration in parieto-occipital regions ($p < .05$). Three clusters (right frontal, left fronto-temporal, and left centro-parieto-occipital cortex, all $p < .05$) showed increased slow-gamma power after 75–125 min (with strongest effects after 100 min) of ayahuasca administration. Fast-gamma power was increased in 4 clusters (right frontal, right parieto-occipital, left fronto-temporal, and left centro-parieto-occipital cortex, all $p < .05$) 75–125 min after ayahuasca intake. DMT plasma levels (and to a lesser extend its metabolites DMT-NO, NMT, and 3-IAA) showed mostly positive correlations (except for negative correlations with alpha power) with EEG power in the beta, gamma, and delta bands. Similar correlations were found with the β-carbolines harmine, harmaline and THH.
Sanches et al. 2016 [10]	Open-label SPECT study with 2 sessions per subject: one baseline scan before and one post-acute scan 8 hours after ayahuasca intake on the same day.	17 depressed patients (14 female, mean age 43 years).	2.2 mL/kg ayahuasca (120-200 mL); the ayahuasca batch contained 0.8 mg/mL DMT, 0.21 mg/mL harmine.	[^{99m}Tc]-ECD SPECT	Ayahuasca intake resulted in significant reductions in depressive symptoms (HAM-D and MADRS scales) between 80 to 180 minutes after intake ($p < .01$) and from day 1 to day 21 ($p < .001$). Cerebral blood perfusion notably increased ($p < .01$, corrected) in the left nucleus accumbens (NAc), right insula, and left subgenual area, regions previously found to be hypoactive in depressed patients. This

					increase in blood perfusion is associated with antidepressive effects and contrasts with findings from acute scans, suggesting potential specificity to depressed patients or the post-acute ayahuasca state.
Valle et al. 2016 [11]	Double-blind, placebo-controlled, randomized, cross-over clinical trial with 4 experimental sessions. Participants received placebo+placebo, placebo+ayahuasca, ketanserin+placebo and ketanserin+ayahuasca on 4 different occasions. Participants received first either placebo or ketanserin and one hour later either placebo or lyphilized ayahuasca capsules. EEG recordings were collected before administration of placebo or ketanserin and 90 min after placebo or ayahuasca administration.	12 healthy subjects (5 female, mean age 35 years) with prior experience with psychedelic substances.	lyophilized ayahuasca capsules (0.75 mg/kg DMT, 1.51 mg/kg harmine, 0.16 mg/kg harmaline and 1.33 mg/kg THH) and 40 mg ketanserin.	EEG; resting-state with closed eyes	Ayahuasca led to decreased delta power ($p < .05$) in the occipital and parietal cortex, decreased theta ($p < 0.05$) and alpha power ($p < .01$) on the whole scalp 90 min after administration. No changes were found for the beta band. Frequency variability was found to be increased after ayahuasca as measured by centroid deviation. Ketanserin only treatment showed opposite effects than ayahuasca with significantly increased in delta and theta power and no changes in alpha power. For combined treatment with ketanserin+ayahuasca, these effects were amplified, showing stronger increases in delta and theta power and reductions in centroid deviation. Ayahuasca additionally led to current density decreases in the alpha band in the posterior brain, in the theta band in lateral and medial frontal lobe, and in delta band in the inferior temporal gyrus. Ketanserin alone did not lead to changes in current density, and ketanserin+ayahuasca again led to opposite changes than ayahuasca alone with current density increases in the delta and theta band. Combining ketanserin+ayahuasca led to significantly lower subjective changes than ayahuasca alone but did not block all subjective effects completely.
Sampedro et al. 2017 [12]	Open-label fMRI study without control group. [^{1}H]-MRS and resting-state fMRI scans were conducted once at baseline up to 24 hours before drug administration and once in the post-acute phase up to 24 hours after ingestion. Three volumes of interest (VOI) were measured with MRS, i.e. PCC, ACC, and cerebellum.	16 healthy subjects (6 female, mean age 39 years) with prior ayahuasca experience.	Mean dose of ayahuasca was 148 ± 29 mL; corresponds to 45 ± 9 mg DMT, 126 ± 25 mg harmine, 26 ± 5 mg tetrahydroharmine, and 5 ± 1 mg harmaline.	[^{1}H]-MRS and rs-fMRI with closed eyes	MRS: Three volumes of interest (VOI) were measured with MRS, i.e. PCC, ACC, and cerebellum. Post-acute decreases in glutamate+glutamine ($p < .05$, uncorrected), creatinine+phospho-creatinine ($p < .05$, FDR-corrected), and N-acetylaspartate+N-acetylaspartylglutamate ($p < .05$, FDR-corrected) levels were found in the PCC, but no significant changes were found in the other two VOIs.<br>fMRI: For resting-state fMRI (all results are $p < .05$, FWE-corrected), 3 VOIs (1 in PCC, 2 in ACC) were selected for subsequent analyses. PCC: post-acute increased connectivity to cuneus, ACC, pre- and post-central gyrus, superior temporal gyrus and insula. ACC: post-acute increased connectivity to precuneus, right parahippocampal gyrus, hippocampus and amygdala, left angular gyrus and inferior parietal lobule, and PCC; decreased connectivity to cuneus.
Viol et al. 2017 [13]	Same study design as in de Araujo et al. 2012, only resting-state data was considered for this paper.	Same sample as in de Araujo et al. 2012, but only 7 included in analysis.	Same dose as in de Araujo et al. 2012.	fMRI; resting-state with closed eyes	ayahuasca intake led to significant changes in 16 brain networks, resulting in higher degree variance and lower kurtosis in individual networks, which was interpreted as being indicative of an increased Shannon entropy. Specific calculations of Shannon entropy found significant increases in Shannon entropy for each subject. Global integration as calculated by mean geodesic distance (increased after ayahuasca) and global efficiency (decreased after ayahuasca) was significantly decreased for every subject at the single-subject as well as at the group level. Local integration as calculated with clustering coefficients and local efficiencies (both parameters were increased after ayahuasca) was significantly increased after ayahuasca.
Pasquini et al. 2020 [14]	Randomized, double-blind, placebo-controlled, between-subject fMRI study. Subjects underwent a baseline fMRI scan one day prior and a post-acute fMRI scan one day after drug administration.	43 healthy subjects (23 female, mean age 31 years) with no prior ayahuasca experience.	1.0 mL/kg ayahuasca; the ayahuasca batch contained 0.36 mg/mL DMT, 1.86 mg/mL harmine, 0.24 mg/mL harmaline, 1.20 mg/mL THH.	fMRI; resting-state	The authors investigated within- and between-network functional connectivity changes after ayahuasca administration in the salience, default mode, visual, and sensorimotor network. For the contrast of post-acute - baseline measurements, results showed increased ACC connectivity within the salience network and decreased connectivity in the PCC within the DMN; and increased connectivity between DMN and salience network in the ayahuasca compared to the placebo group.
Simonsson et al. 2022 [15]	Same study design as in Bouso et al. 2015.	Same sample as in Bouso et al. 2015.	No dose was administered within the study.	structural MRI	Given the *a priori* hypothesis, only the corpus callosum was compared between ayahuasca and matched group. The ayahuasca group showed a significantly thicker ($p < .01$) than the control group. There was a significant positive correlation of times of ayahuasca use and rostral corpus callosum ($p < .05$) that did not survive corrections for multiple comparisons.
Sanchez et al. 2024 [16]	Open-label, subjects underwent two imaging sessions before and 50 min after ayahuasca administration.	19 healthy male subjects (mean age 32 years).	25–35 mL of ayahuasca, depending on the extend of prior experience; corresponds to a mean dose of 0.33 mg/kg DMT, 0.47	fMRI; implicit aversive stimulation task	Participants reported less anxiety and more sedation (Visual analog scales, $p < .01$, Bonferroni-corrected) after ayahuasca intake compared to baseline and showed longer reaction times ($p < .05$) to aversive than to neutral images, this effect vanished after ayahuasca administration. Bilateral amygdala showed reduced ($p < .05$, FDR-corrected) reactivity to aversive stimuli after ayahuasca

			mg/kg harmine, 0.02 mg/kg harmaline, and 0.47 mg/kg THH		consumption compared to baseline. Exploratory results showed increased activation of the insula bilaterally, and of the right dorsolateral prefrontal cortex.
Mallaroni et al. 2023 [17]	Cross-sectional study comparing T1-weigthed MR images of frequent ayahuasca users with a matched control group derived from an online MR repository.	24 healthy members of the Santo Daime church (10 female, mean age 55 years) and 24 for age and sex matched controls (10 female, mean age 55 years).	No dose was administered within the study.	structural MRI	Groups were compared with morphometric similarity network (MSN) analysis. The ayahuasca group showed an overall reduced mean morphometric similarity (MS) than the control group ($p < .0001$). Regions of high positive MS were predominantly found in frontal and temporal cortices, while regions of high negative MS were overall located in occipital and motor cortices. The ayahuasca group showed decreased MS in sensorimotor cortices (inferior frontal gyrus, precuneus, pre and post central gyrus) and increased MS in midline, temporal and prefrontal structures (orbitofrontal, entorhinal, cingulate, and insular cortices). In the ayahuasca group, more regions showed architectonic differentiation (i.e., uncoupling) that de-differentiation, with 56% and 30% of the analyzed regions, respectively. Clustering the analyzed regions into the 7 functional RSN defined by Yeo et al. [18] resulted in reduced MS in the ayahuasca group in the SM, DAN and DMN, and increased MS in LIM. Clustering the analyzed regions into the anatomically corresponding Von Economo cytoarchitectonic organization atlas [19] revealed decreased MS in granular association isocortical classes 1 and 2, and increased MS in the insular and limbic classes (all $p < .001$). Gene expression analyses of genes relevant for DMT's or ayahuasca's mechanisms of action showed correlations between regions with overexpression of those genes and regions with increased MS (positive correlation) and negative MS (negative correlation) in the ayahuasca – control contrast. 18 of the 66 investigated genes showed significant contributions in the partial least squares regression used; 11 genes were overexpressed in high MS regions and 7 were under-expressed in low MS regions.
Mallaroni et al. 2024 [20]	Open-label, fixed-order, within-subject fMRI study with 2 experimental sessions on two consecutive days. Groups of 6 Santo Daime members conducted the study days together and either waited (on the first no-drug day) and were scanned 90 min after their turn or ingested ayahuasca (on the second day) in turns and were scanned 90 min after ayahuasca administration.	21 healthy members of the Santo Daime church (10 female, mean age 54 years).	Participants self-administered a volume of ayahuasca corresponding to their usual dose (mean 24 mL with concentrations of 0.14 mg/mL DMT, 4.5 mg/mL harmine, 0.51 mg/mL harmaline, and 2.10 mg/mL THH)	fMRI, resting-state with closed eyes	Ayahuasca ingestion increased all subscale scores of the 5D-ASC and participants reported more internal singing while in the MR scanner under the influence of ayahuasca compared to placebo. Connectome fingerprints (assumes that FC should be more similar between test-retest scans of the same subject than between different subjects) were calculated with "identifiability matrices" to reflect both inter- and intra-individual variability of the functional connectome. Static identifiability measures showed a decreased differential identifiability after ayahuasca ingestion, meaning that participant connectomes mirrored each other. Repeating the same analysis on shorter temporal scales (dynamic calculation) resulted in the same pattern as with the static approach. Static within-network analyses of 7 RSNs revealed reduced network stability in the SAL and increased stability in the DAN after ayahuasca. Between-network stability between each combination of network pairs revealed mostly reductions in stability arising from the SAL and VIS. Dynamic connectomes revealed changing connectivity patterns over time, where the DMN, the SAL and the DAN showed decreased within-network edge stability and the VIS showed greater stability. Between-network edge stability reductions were found in many network pairs.

DMT

Daumann et al. 2008 [21]	Randomized, double-blind, placebo-controlled, within-subject, DMT or placebo was administered soon after participant's arrival, the other condition was conducted 2 h after the first condition.	14 healthy subjects (8 men; mean age 32.1 years).	I.v. DMT, bolus: 0.15 mg/kg over 5 min, 1 min break, then continuous infusion of 0.01-0.01875 mg/(kg × min) for 20 min.	fMRI; covert orienting of attention task	DMT induced increased reaction time to the stimuli compared to placebo in every task condition ($p < .05$, corrected), while between condition reaction time during DMT did not differ anymore, indication a blunting of the inhibition of return (IOR) phenomenon under the influence. No difference in fMRI data between placebo and DMT contrast was found.

Heekeren et al. 2008 [22]	Randomized, double-blind, cross-over design with 4 individual dosing sessions, one low and one high dose of DMT and one low and one high dose of *S*-ketamine. 20 min after start of the infusions, 30 min of EEG was recorded during a mismatch negativity (MMN) task were recorded. EEG recordings of the MMN task were also measured on a separate baseline day before the first dosing session.	15 healthy volunteers (6 women, mean age 38 years). Only 9 subjects completed all 4 dosing sessions and were included in the analysis.	Low dose DMT: bolus injection over 5 min with 0.15-0.2 mg/kg, 1 min break, then continuous infusion with 11.3–15 µg/kg per min over 84 min. High dose DMT: bolus injection over 5 min with 0.2-0.3 mg/kg, 1 min break, then continuous infusion with 15–20 µg/kg per min over 84 min.	EEG; mismatch negativity (MMN) with auditory stimulation and a simultaneous cognitive performance task	*S*-ketamine results are not considered here. Task performance during the MMN auditory stimulation decreased for both low and high dose DMT compared to baseline. N1 peak amplitude was lower for in the low dose DMT session ($p < .01$), but not for the high dose condition compared to baseline. Amplitude latencies did not show changes for both drug conditions. MMN activity only showed significant reductions in 1 of 4 signal sources located at the right temporal pole for the low-dose condition compared to baseline.
Daumann et al. 2010 [23]	Same study design as in Daumann et al. 2008.	Same sample as in Daumann et al. 2008.	Same dose as in Daumann et al. 2008.	fMRI; target-detection task	Reaction time to auditory and visually cued stimuli increased under the influence of DMT compared to placebo ($p < .01$), and the benefit of a cues was smaller compared to the placebo condition. Analysis of cue benefit in the DMT condition showed a lesser reduction in RT ($p < .01$) to the sensory cues, indicating greater difficulty in reacting to environmental stimuli Neuroimaging analysis revealed smaller activations in regions associated with the processing of visual cues, such as the left inferior and right middle occipital gyrus, the right inferior temporal gyrus, the right cuneus and the right culmen, and one region associated with processing of auditory cues, i.e. the right middle temporal gyrus.
Timmermann et al. 2019 [24]	Open-label fixed-order, within-subject, placebo-controlled design. Placebo was administered in the first of two sessions, DMT in the second session one week later. EEG recordings were conducted from 1 min prior to injection to 20 min after injection.	13 healthy participants (6 female, mean age 34 years) with prior psychedelic experiences.	Placebo (saline) injection at first session, intravenous DMT at second session 1 week later was administered over 30 s in 4 different doses increasing with progress of study to control for safety; three participants received 7 mg, four received 14 mg, one received 18 mg and 5 received 20 mg.	EEG; task free with intensity ratings and closed eyes	DMT led to significant reductions in alpha power and posterior beta power while increasing signal diversity and delta and gamma power. Intensity ratings showed positive correlations with increased delta power and signal diversity and negative correlations with global alpha and posterior beta power changes. DMT also led to reductions on backward wave and increased forward wave power. Signal diversity showed strong positive correlation with "richness of the experience".
Alamia et al. 2020 [25]	Same study design as in Timmermann et al. 2019.	Same sample as in Timmermann et al. 2019.	Same dose as in Timmermann et al. 2019.	EEG; task free with intensity ratings and closed eyes	Cortical travelling waves (i.e., spatio-temporal patterns of cortical activations in two directions: forward waves (FW) and backward waves (BW)) were assessed under the influence of DMT and placebo. FW waves significantly increased 5 min after DMT injection compared to before injection and compared to placebo injection. BW significantly increased after placebo administration compared to before, while DMT administration led to a decrease in BW compared to baseline and compared to placebo. The different dosages used in this study did not confound the results. The frequency of travelling waves before administration of either placebo or DMT showed strong alpha-range oscillatory rhythm, which after DMT administration changed: alpha and beta band oscillations decreased, and delta and theta band oscillations increased for BW and FW.
Pallavicini et al. 2021 [26]	Open-label study in naturalistic setting. Subjects underwent baseline EEG recordings (5 min with open eyes, 5 min with closed eyes) before DMT administration and from the timepoint of DMT administration until participants reported being back at baseline (6 ± 1.4 min).	35 healthy participants (7 female, mean age 33 years) with previous ayahuasca or DMT experience.	No DMT was not provided by the researchers, subjects either attended ceremonies or retreats; DMT was administered via inhalation.	EEG; task free, open and closed eyes (closed eyes during DMT effects)	13 out of 35 (37%) participants reported a "complete mystical-type experiences" according to the MEQ30, highest scores in the 5D-ASC were reported for the three subscales elementary imagery, complex imagery, and audio-visual synesthesia. EEG analysis (all results are presented with $p < .05$, FDR-corrected) showed reductions in alpha waves at 10 Hz in the DMT condition compared to baseline (closed eyes) for all electrodes. DMT additionally led to significant increases in low (< 3 Hz) and high (> 36 Hz) in occipital, parietal, antero-central and temporal regions. Time frequency analysis indicated a smooth temporal evolution of average alpha power that returned to baseline levels from the timepoint of DMT administration until 7 min after. Alpha power was significantly decreased after DMT administration for ~3 min. Coherence and metastability of the EEG signal was increased in the gamma band and decreased in the alpha band for the contrast DMT > baseline (closed eyes), signal complexity was increased in all channels in the DMT condition.

Tagliazucchi et al. 2021 [27]	Same study design as in Pallavicini et al. 2021.	Same sample as in Pallavicini et al. 2021.	Same dose as in Pallavicini et al. 2021.	EEG; task free, open and closed eyes (closed eyes during DMT effects)	All results are FDR-corrected with a $p < .05$. Acute DMT recordings showed posterior increases in delta and theta power, decreases in alpha power in occipito-temporal regions, beta power increased in posterior and decreased in prefrontal regions, gamma power increased in occipital, parietal and temporal regions. Correlations of psychometric scales (subscale "Experience of Unity" from 5D-ASC, and subscales "Mystical Experience" and "Transcendence of Time and Space" from the MEQ30) showed a negative correlation with theta power changes.
Timmermann et al. 2023 [28]	Pseudo-randomized, single-blind, placebo-controlled, within-subject design, DMT and placebo were administered intravenously and participants while already being in the fMRI scanner (with simultaneous EEG measurements) for 8 minutes. Scanning continued for 20 minutes.	20 healthy participants (7 female, mean age 34 years) with previous psychedelic experiences were recruited, not all were included in analysis (exact number depends on imaging modality).	20 mg DMT or placebo (saline) injected over 30 s.	fMRI + EEG; both task free with intensity ratings	Real-time intensity ratings reached peak mean values of 8 out of 10 3–4 min after DMT administration and remained significantly higher than placebo until 17 min after injection.<br>fMRI: Within-network analyses of 7 RSN defined by Yeo et al. [18] showed reductions ($p < .05$, FDR-corrected) in network integrity for the VIS, SM, DAN, FP, and DMN and increased within-network global function connectivity for the SAL, FP, and DMN (all at least $p < .01$, FDR-corrected) in the DMT condition. Between-network separation was reduced the FP, DMN and SAL and other networks, while overall global functional connectivity especially increased for networks processing higher cognitive functions during DMT effects. Dynamic functional connectivity changes showed significant correlations between the real-time intensity ratings. The principal gradient (with primary sensory and motor information processing on one end and integration and computation of higher cortical functions on the other end) was significantly reduced during the acute effects of i.v. DMT.<br>EEG: Total and oscillatory spectral power in the alpha and beta bad was significantly reduced after DMT administration, while fractal spectral power was reduced for all frequencies below 30 Hz after DMT injection. Signal diversity showed increases after DMT administration compared to placebo. Negative Correlations could be seen between real-time intensity ratings and power in alpha and beta bands, while positive correlations between intensity and delta and theta power in the oscillatory component were observed. Higher signal diversity showed positive correlation with intensity ratings. Very similar patterns were seen for correlations between plasma DMT concentrations and EEG measures, as well as for neurophenomenological assessments (intensity of visuals, body load and emotional/metacognitive changes) and EEG outcomes.<br>Several correlations between EEG changes and fMRI resting state functional connectivity were found: frontal delta power showed positive correlations with GFC in 6 out 7 RSNs, alpha power in parietal regions was negatively correlated with GFC in 5 out of 7 RSNs, occipital gamma power showed positive correlations with GFC in 2 RSNs and signal diversity correlated positively with GFC in 3 out of 7 RSNs.
Eckernäs et al. 2023 [29]	Same study design as in Timmermann et al. 2019.	Same sample as in Timmermann et al. 2019.	Same dose as in Timmermann et al. 2019.	EEG; task free with intensity ratings and closed eyes	After visual inspection, correlations between PK parameters (DMT concentrations at various timepoints up to 20 min after DMT administration) and alpha and beta power and signal complexity were selected for further dose-dependent PKPD modelling. The final models were used to simulate the expected effects on alpha and beta power and signal complexity at six different DMT doses (1, 4, 7, 10, 14 and 20 mg) in 100 subjects. The results of the simulations revealed a dose-dependent decrease in alpha and beta power (higher dose leads to stronger decrease) and a dose-dependent increase in signal complexity (higher dose leads to higher increase). Relationships between plasma concentrations and effects on EEG signal were described with effect compartment models and indicated plasma DMT concentrations of 71 nM for the alpha band, 137 nM for the beta band, and 54 nM for signal complexity to reach half of the maximum response (IC_{50} and EC_{50}).

Abbreviations: DMT = *N,N*-dimethlytryptamine, THH = tetrahydroahrmine, ACC = anterior cingulate cortex, PCC = posterior cingulate cortex, (m)PFC = (medial) prefrontal cortex, MFG = left middle frontal gyrus, MTG = middle temporal gyrus, IPL = inferior parietal lobule, HRS = hallucinogen rating scale, MMN = mismatch negativity, EEG = electroencephalography, (f)MRI = (functional) magnetic resonance imaging, BOLD = blood oxygenation level dependent, MRS = magnetic resonance spectroscopy, [^{99m}Tc]-ECD SPECT = technetium-labeled ethyl cysteinate dimer single photon emission computed tomography, 5D-ASC = 5-dimensions altered states of consciousness questionnaire, MEQ30 = mystical experience questionnaire, MS = morphometric similarity, MSN = morphometric similarity network, DMN = default mode network, SAL = salience network, SM = sensory-motor network, DAN = dorsal attention network, FPN = fronto-parietal network, VIS = visual network, LIM = limbic network, RSN = resting state network

References

1. Don NS, McDonough BE, Moura G, et al (1998) Effects of Ayahuasca on the human EEG. Phytomedicine 5:87–96. https://doi.org/10.1016/S0944-7113(98)80003-2

2. Riba J, Anderer P, Morte A, et al (2002) Topographic pharmaco-EEG mapping of the effects of the South American psychoactive beverage ayahuasca in healthy volunteers. Br J Clin Pharmacol 53:613–628. https://doi.org/10.1046/j.1365-2125.2002.01609.x

3. Riba J, Anderer P, Jané F, et al (2004) Effects of the South American Psychoactive Beverage Ayahuasca on Regional Brain Electrical Activity in Humans: A Functional Neuroimaging Study Using Low-Resolution Electromagnetic Tomography. Neuropsychobiology 50:89–101. https://doi.org/10.1159/000077946

4. Riba J, Romero S, Grasa E, et al (2006) Increased frontal and paralimbic activation following ayahuasca, the pan-Amazonian inebriant. Psychopharmacology (Berl) 186:93–98. https://doi.org/10.1007/s00213-006-0358-7

5. Alonso JF, Romero S, Mañanas MÀ, Riba J (2015) Serotonergic Psychedelics Temporarily Modify Information Transfer in Humans. International Journal of Neuropsychopharmacology 18:. https://doi.org/10.1093/ijnp/pyv039

6. De Araujo DB, Ribeiro S, Cecchi GA, et al (2012) Seeing with the eyes shut: neural basis of enhanced imagery following Ayahuasca ingestion. Hum Brain Mapp 33:2550–2560. https://doi.org/10.1002/HBM.21381

7. Palhano-Fontes F, Andrade KC, Tofoli LF, et al (2015) The psychedelic state induced by ayahuasca modulates the activity and connectivity of the default mode network. PLoS One 10:e0118143. https://doi.org/10.1371/journal.pone.0118143

8. Bouso JC, Palhano-Fontes F, Rodriguez-Fornells A, et al (2015) Long-term use of psychedelic drugs is associated with differences in brain structure and personality in humans. European Neuropsychopharmacology 25:483–492. https://doi.org/10.1016/j.euroneuro.2015.01.008

9. Schenberg EE, Alexandre JFM, Filev R, et al (2015) Acute Biphasic Effects of Ayahuasca. PLoS One 10:e0137202. https://doi.org/10.1371/journal.pone.0137202

10. Sanches RF, de Lima Osorio F, Dos Santos RG, et al (2016) Antidepressant Effects of a Single Dose of Ayahuasca in Patients With Recurrent Depression: A SPECT Study. J Clin Psychopharmacol 36:77–81. https://doi.org/10.1097/JCP.0000000000000436

11. Valle M, Maqueda AE, Rabella M, et al (2016) Inhibition of alpha oscillations through serotonin-2A receptor activation underlies the visual effects of ayahuasca in humans. European Neuropsychopharmacology 26:1161–1175. https://doi.org/https://doi.org/10.1016/j.euroneuro.2016.03.012

12. Sampedro F, de la Fuente Revenga M, Valle M, et al (2017) Assessing the Psychedelic "After-Glow" in Ayahuasca Users: Post-Acute Neurometabolic and Functional Connectivity Changes Are Associated with Enhanced Mindfulness Capacities. Int J Neuropsychopharmacol 20:698–711. https://doi.org/10.1093/ijnp/pyx036

13. Viol A, Palhano-Fontes F, Onias H, et al (2017) Shannon entropy of brain functional complex networks under the influence of the psychedelic Ayahuasca. Sci Rep 7:7388. https://doi.org/10.1038/s41598-017-06854-0

14. Pasquini L, Palhano-Fontes F, Araujo DB (2020) Subacute effects of the psychedelic ayahuasca on the salience and default mode networks. J Psychopharmacol 34:623–635. https://doi.org/10.1177/0269881120909409

15. Simonsson O, Bouso JC, Kurth F, et al (2022) Preliminary evidence of links between ayahuasca use and the corpus callosum. Front Psychiatry 13:. https://doi.org/10.3389/fpsyt.2022.1002455

16. Arruda Sanchez T, Ramos LR, Araujo F, et al (2024) Emotion regulation effects of Ayahuasca in experienced subjects during implicit aversive stimulation: An fMRI study. J Ethnopharmacol 320:117430. https://doi.org/10.1016/j.jep.2023.117430

17. Mallaroni P, Mason NL, Kloft L, et al (2023) Cortical structural differences following repeated ayahuasca use hold molecular signatures. Front Neurosci 17:1217079. https://doi.org/10.3389/fnins.2023.1217079

18. Yeo BTT, Krienen FM, Sepulcre J, et al (2011) The organization of the human cerebral cortex estimated by intrinsic functional connectivity. J Neurophysiol 106:1125–65. https://doi.org/10.1152/jn.00338.2011

19. Scholtens LH, de Reus MA, de Lange SC, et al (2018) An MRI Von Economo – Koskinas atlas. Neuroimage 170:249–256. https://doi.org/10.1016/j.neuroimage.2016.12.069

20. Mallaroni P, Mason NL, Kloft L, et al (2024) Shared functional connectome fingerprints following ritualistic ayahuasca intake. Neuroimage 285:120480. https://doi.org/10.1016/j.neuroimage.2023.120480

21. Daumann J, Heekeren K, Neukirch A, et al (2008) Pharmacological modulation of the neural basis underlying inhibition of return (IOR) in the human 5-HT2A agonist and NMDA antagonist model of psychosis. Psychopharmacology (Berl) 200:573–583. https://doi.org/10.1007/s00213-008-1237-1

22. Heekeren K, Daumann J, Neukirch A, et al (2008) Mismatch negativity generation in the human 5HT2A agonist and NMDA antagonist model of psychosis. Psychopharmacology (Berl) 199:77–88. https://doi.org/10.1007/s00213-008-1129-4

23. Daumann J, Wagner D, Heekeren K, et al (2010) Neuronal correlates of visual and auditory alertness in the DMT and ketamine model of psychosis. Journal of Psychopharmacology 24:1515–1524. https://doi.org/10.1177/0269881109103227

24. Timmermann C, Roseman L, Schartner M, et al (2019) Neural correlates of the DMT experience assessed with multivariate EEG. Sci Rep 9:16324. https://doi.org/10.1038/s41598-019-51974-4

25. Alamia A, Timmermann C, Nutt DJ, et al (2020) DMT alters cortical travelling waves. Elife 9:. https://doi.org/10.7554/eLife.59784

26. Pallavicini C, Cavanna F, Zamberlan F, et al (2021) Neural and subjective effects of inhaled N,N-dimethyltryptamine in natural settings. Journal of Psychopharmacology 35:406–420. https://doi.org/10.1177/0269881120981384

27. Tagliazucchi E, Zamberlan F, Cavanna F, et al (2021) Baseline Power of Theta Oscillations Predicts Mystical-Type Experiences Induced by DMT in a Natural Setting. Front Psychiatry 12:. https://doi.org/10.3389/fpsyt.2021.720066

28. Timmermann C, Roseman L, Haridas S, et al (2023) Human brain effects of DMT assessed via EEG-fMRI. Proceedings of the National Academy of Sciences 120:. https://doi.org/10.1073/pnas.2218949120

29. Eckernäs E, Timmermann C, Carhart-Harris R, et al (2023) N,N-dimethyltryptamine affects electroencephalography response in a concentration-dependent manner—A pharmacokinetic/pharmacodynamic analysis. CPT Pharmacometrics Syst Pharmacol 12:474–486. https://doi.org/10.1002/psp4.12933
